# Risk factors affecting dairy cattle protective grouping behavior, commonly known as bunching, against *Stomoxys calcitrans (L*.*)* on California dairies

**DOI:** 10.1371/journal.pone.0224987

**Published:** 2019-11-07

**Authors:** Wagdy R. El Ashmawy, Deniece R. Williams, Alec C. Gerry, John D. Champagne, Terry W. Lehenbauer, Sharif S. Aly

**Affiliations:** 1 Veterinary Medicine Teaching and Research Center, School of Veterinary Medicine, University of California Davis, Tulare, California, United States of America; 2 Department of Medicine and Infectious Diseases, Faculty of Veterinary Medicine, Cairo University, Giza, Egypt; 3 Department of Entomology, University of California, Riverside, California, United States of America; 4 Department of Population Health and Reproduction, School of Veterinary Medicine, University of California, Davis, California, United States of America; University of Illinois, UNITED STATES

## Abstract

Bunching is the term used to describe the protective aggregating behavior of cattle against the painful bites of stable flies (*Stomoxys calcitrans*), where cattle gather in a group with their heads to the center of the group and their tails to the outside to reduce stable fly attack. Both sexes of the stable fly feed on blood, and their painful bites negatively impact cattle health, productivity and welfare. A longitudinal study was conducted from April to July 2017 to estimate the stable fly activity on 20 California dairies (average herd size = 2466 ± 1050), to determine stable fly activity that induced bunching, and to evaluate the association between management and environmental factors, and cattle bunching. Stable fly activity was recorded weekly using trap counts and leg counts. Data was analyzed using linear mixed models with odds ratio. Cattle bunching at the dairy level was predicted by mean trap counts of ≥150 flies/trap/week, while bunching at the pen level was predicted by mean leg counts >1 fly/leg/cow or mean trap counts >50 flies/trap/week for traps closest to the pen. Additional risk factors predicting cattle bunching at the dairy level were study week (May/June vs July), presence of crops adjacent to dairy >2 sides, and feeding wet distillers grain. Additional risk factors predicting cattle bunching at the pen level were study week (May/June vs July), ambient temperature ≤30°C, pen design (freestall vs open dry lot or bedded pack), production status (lactating/dry vs close-up), presence of crops surrounding cattle pens, feeding rations containing molasses. Cattle bunching was reduced at the pen level by relative humidity >50%, and when the cattle pen was surrounded by other cattle pens or was bordered by a main road. At the dairy level, removal of manure along fence lines of cattle pens was protective against cattle bunching.

## Introduction

Stable flies (*Stomoxys calcitrans)* are biting flies that feed on blood leading to high stress and elevated cortisol levels which may impact cattle health, productivity and welfare [1]. Previous studies have reported severe reduction in US dairy and beef cattle productivity due to *S*. *calcitrans* [2–7]. In 2009, the losses due to stable flies in the U.S. cattle industry were estimated to be $2.2 billion/year, with $360 million losses in the dairy industry [8]. Stable flies tend to bite the lower parts of the body such as the legs and abdomen, irritating cattle and provoking individual or group behavioral changes to repel or avoid the fly attack. Cattle might differ in how aggressively they engage in fly-repelling behaviors according to their breed, color, parity, productivity, and the number of stable flies attacking them [9–11]. Fly-repellent behaviors include tail flicking, foot stomping, head tossing, skin twitching, and ear trembling to reduce the fly attack [1, 9, 10, 12]. Additionally, cattle tend to aggregate in a tight group with their heads to the center of the group and their tails to the outside to protect themselves against the stable fly attack displaying a behavior known as bunching [12–15].

Bunching may protect cattle positioned within the group from stable fly bites due to the encounter-dilution effect in which the number of flies per cow decreases with increasing group size of the bunch [16, 17]. However, bunching can increase the risk of traumatic injuries and heat stress as cows seek less exposure to biting flies in the center compared to at the periphery of the cow bunch [18]. Furthermore, bunching can decrease cattle resting and feeding [13] ultimately reducing milk production and milk butter fat content due to the associated reduction in dry matter intake and rumination time.

Seasonal activity and intensity of stable flies commonly peak during the spring and early summer [6, 19]. Fly abundance depends on the amount of rain in early spring and the ambient temperature throughout spring and summer[20]. Impact of stable fly abundance on production in cattle varies by fly intensity. Studies on beef cattle showed that high stable fly numbers are associated with reduced average daily weight gain [7, 21]. Likewise, studies on dairy cattle found an inverse association between the number of stable flies and quantity and quality of milk production[22]. Bishop [2] reported a decrease in milk production of 40 to 60% due to stable fly attack in Texas and Oklahoma. Freeborn *et al*., [3] reported a reduction of 9.2% in milk production due to stable fly attack. Bruce and Decker [5] estimated the reduction in butter fat as a result of stable flies as 0.65%/fly/cow. Berry *et al*., [23] reported an estimated loss in milk production of 2.3 and 3.51 kg/ cow/ day during summer of 1980 and 1981, respectively. Taylor *et al*. [8] estimated the median annual milk losses of 139 kg /cow /year due to stable flies [3–6, 23]. However, dairy facility design and management practices have changed drastically since these earlier studies which may have introduced new risk factors for stable fly abundance and bunching.

Finally, dairy producers’ report large variation of bunching both between and within dairies, the reasons for which remain unknown and, as such, recommendations for producers to minimize cattle bunching behavior are lacking. Hence, our study hypothesized that bunching by dairy cows is induced as a protective aggregative behavior at some threshold of stable fly activity; and that initiation of this behavior is affected by the environment, animal and facility management, and other cow-related factors.

In this study we modeled the number of stable flies either per cow leg or per fly trap associated with bunching behavior of cows. We also examined the effect of environmental and management factors on California dairy farms that were associated with cattle bunching behavior.

## Materials and methods

The current research has been approved by University of California Davis Institutional Animal Care and Use Committee (protocol number 19088) and exempted by the Institutional Review Board (protocol number 1476320–1).

### Study herds

A convenience sample of twenty California dairies were enrolled, 19 in Tulare County and one in Kings County. Selection of dairies to participate in the study was based on owner willingness to participate, location, herd size, and variation in pen designs (freestall, open lot and mixed), cow breed (Holstein or Jersey) and fly control measures used. Dairies were located near the city of Tulare and therefore in proximity to most of the geographic and anthropological landmarks in the region such as riparian areas, waterways, foliage, crops, highways, railroad tracks, landfill and water reservoirs.

### Study design

A longitudinal study was conducted between April 26^th^, 2017 and July 31^st^, 2017. The study commenced with an on-farm enrollment survey (S1 File) that included a walk-through component to record the environmental factors and management practices on each study dairy.

**Enrollment and bunching risk factor survey**. An in-person survey with the herd owner or manager on each study dairy was completed prior to start of the study in April 2017. The survey included 151 questions to collect information about facility design and management practices. Questions were divided into six sections: herd information; facility design; facility management factors (bedding and manure management, commodity and feed bunk management and cow cooling); calf management; history of bunching; and fly control. On each study dairy, the facility design was recorded for lactating, dry and close-up pens, with pen type recorded as freestall (with or without exercise area), dry lot or bedded pack. In addition, the survey included a walk through to record information about cattle housing, cooling system, waste management, and commodities management. Specifically, information recorded in each pen included height and width of the shade and/or the barn roof, methods of cow cooling including use of soakers, water troughs or fans (with notes on functionality), manure accumulation around pen fences, feed curbs, feed refusal, and wet spots, and the presence/absence of leakage around silage storage sites.

**Stable fly counts on fly traps (trap counts).** Five Alsynite traps (Biting Fly Trap®, Olson Products Inc, Medina, OH) were placed on each of the 20 study dairies. Locations of traps on each dairy were determined to optimize trap exposure to the different structures or landmarks on the dairy including trees, roads, fly breeding grounds such as decomposing vegetation (e.g., silage pits or bags where seepage may be expected, manure mounds, and the ends of feed mangers where feed may accumulate). The number and distribution (uniform or biased) of stable flies captured on each Alsynite trap was recorded once per week at all 20 dairies from May 1—July 17, 2017. If the stable fly distribution on the trap was biased, study personnel recorded which side of the trap captured the greatest number of flies and noted nearby facility structures facing that side of the trap. The condition of the trap (good, dusty, broken, tipped over) was also recorded each week and traps were serviced by replacing the Sticky Sleeve^TM^ (Olson Products Inc, Medina, OH). Six trained study personnel serviced the traps and recorded the number of stable flies on each trap. In addition, relative humidity and ambient temperature were recorded for each dairy before counting the first trap using a mobile application called AccuWeather® (American Media Company, State College, Pennsylvania).

**Stable fly counts on cows (leg count).** Stable fly counts on cow legs were determined on 10 of the study dairies selected for variation in dairy location, herd size, pen design, cow breeds and fly control methods used. Two trained study personnel recorded stable fly counts on cow legs. Leg counts were recorded as the number of stable flies on the visible front legs of each cow. Specifically, an observer recorded an instantaneous count of the number of stable flies on the outer side of one forelimb starting from the shoulder down to the hairline above the hoof and the inner side of the other forelimb starting from the chest to the hairline above the hoof using 10 x 42 binoculars (Nikon Prostaff® 3S, Tokyo, Japan) to observe the legs of cattle while the observer stood outside the pen. Leg counts were recorded on cattle that were not bunching and not locked within headstalls so that animals were expressing normal behavior. If there was bunching in the pen such that few cows were outside the bunch, the observer attempted to count flies on cows at the periphery of the bunch. Stable fly leg counts were recorded for 15 cows per pen, and from three different pens at each dairy in accordance with Gerry *et al*. [12]. The count was done using the same pens each week, twice per day, once between 9:00 to 11:00 am and again between 12:00 and 2:00 pm. The observer recorded ambient temperature and relative humidity before every fly count. On one of the study dairies, leg counts were recorded for cattle in 4 pens, a strategic change to allow for estimating the impact of bunching on cow milk production given the cow distribution in pens on that specific dairy; results of this analysis will be reported separately.

#### Cow bunching

Bunching of cows was recorded on each dairy at the same time that leg count and trap counts were recorded. The location of the bunch within the pen was also recorded on a satellite image of the study dairy (Google Earth®, Mountain View, California). Cows particularly in the periphery of the bunch showed signs of leg stomping, tail switching, and skin twitching (S1 Video). The bunching presentation may be altered in freestall pens by the facility design, particularly the presence of structural components including metal bars in the resting areas and limited space in the alleyways. As a result, cattle bunching in a freestall pen was recorded when most of the cows aggregated on one side of the pen and cows in the periphery of the bunch showed signs of stable fly attack such as leg stomping, tail switching and skin twitching.

### Statistical analyses

**Fig 1:**
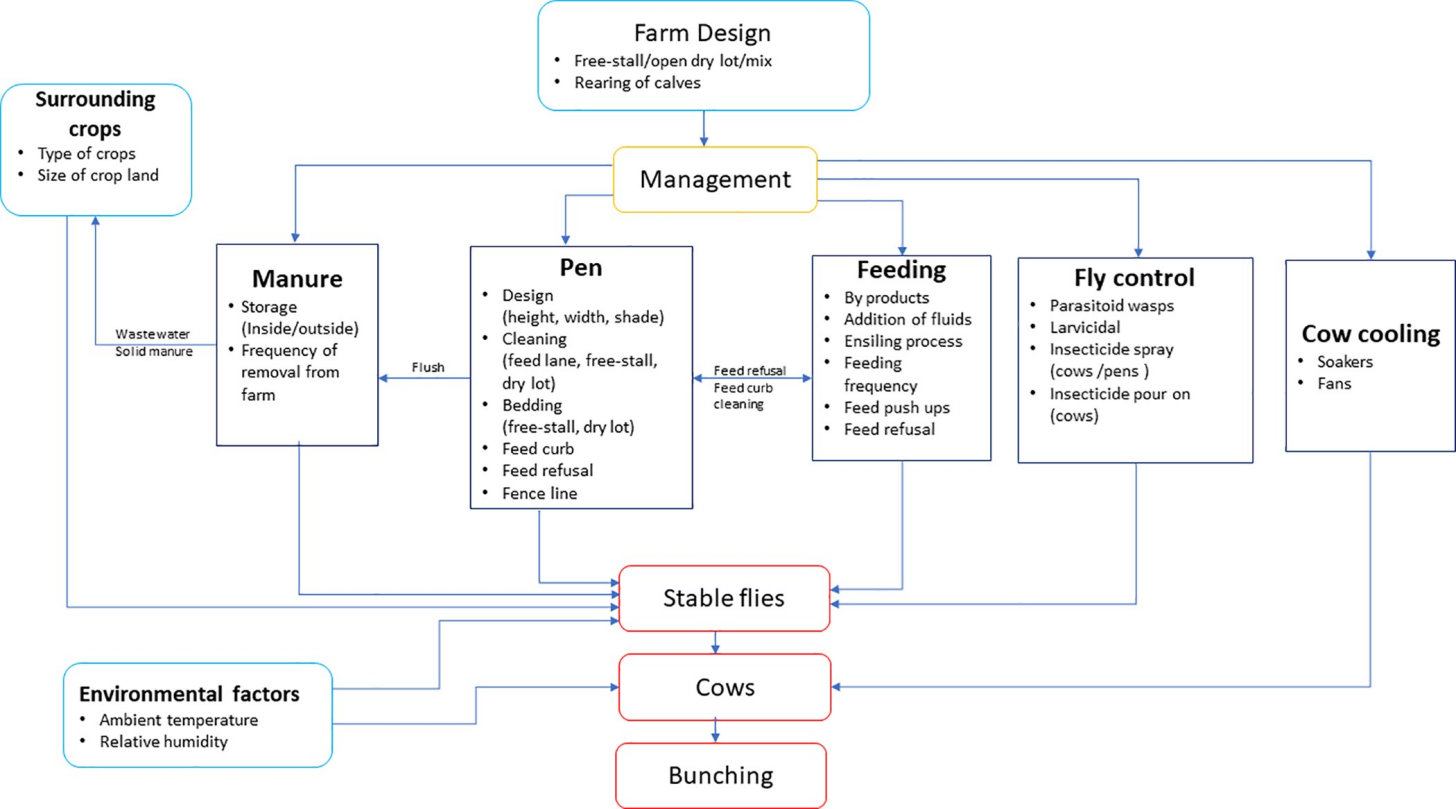
Causal diagram for the explanatory variables that guides the statistical models.

The dichotomous outcome cow bunching recorded as present or absent was modeled using the explanatory variables recorded at both the dairy and the pen level. Fig 1 depicts the causal diagram used to guide the statistical models with the different factors hypothesized to be associated with bunching.

#### Dairy level explanatory variables

*Herd demographics*. The herd profile included cow breed(s), farm design, rolling herd average milk production and whether calves were raised on the premises or not. For dairies that raised their calves on the same premises, we recorded whether calves were raised in individual hutches or groups, and if hutches were raised above the ground with a flush lane beneath them. For hutches with flush lanes, we recorded whether feces accumulated beneath raised hutches was flushed with water, if the flush water was clean or recycled lagoon water, and the frequency of flushing.

*The feeding management of the lactating cows*. Lactating cow variables focused on type of silage storage, feedings per day, frequency of pushing feed in front of mangers, removing refused feed and cleaning the feed curb, and if by-products or fluids (water, molasses) were added to rations.

*Cleaning of the animal pens and manure management*. For freestall facilities, questions focused on the maintenance of the freestall bedding (raking, removal of fecal pats from the freestall, the bedding material and frequency of refilling the freestall beds). For dry-lot (dirt) corrals, questions focused on pen maintenance (raking and scraping), if bedding material was used. For all facilities, manure management variables included type of manure storage, how often solid manure was removed from the dairy, number of recycled flush water lagoons on the dairy, and how often the lagoon water was applied to farmland surrounding the dairy.

*Fly control*. Variables related to fly control methods used included whether the dairy had a fly control program, if they hired a pest control company to provide fly control services and the type and frequency of service (spray the whole dairy, spray the cow legs or supply predatory wasps). If chemicals were used for fly control, the type of chemical, frequency and method of application were also specified as variables.

#### Pen level explanatory variables

These variables were collected during the walk through at enrollment for all the pens on each dairy and before fly counts were recorded. Variables included information on pen design, cow breed, the number of shade structures inside the pen, the height and the width of the shade structure, number of times the cows were milked and the condition and functionality of soakers, water troughs and fans. In addition, the presence of wet spots within the pen, feed refusal, condition of feed curb (clean, dirty), presence of dried manure mounds inside the corrals (in dry lot pens or in the exercise areas of freestall pens), fence line manure build up and the pen surroundings (dairy, crops, trees or main road) as these sites may be associated with development of immature stable flies (19). Variables including mean leg count (flies/leg/cow), relative humidity, ambient temperature and bunching were based on weekly observations as mentioned above.

#### Modeling pen-level cow bunching

A generalized linear mixed model (GLMM) with a logit link was used to model the logit (log odds) of the probability of bunching observed at the pen level on the study dairies using 1) mean stable flies per cow leg (leg count) and 2) the number of stable flies on Alsynite trap close to the pen (trap count). Models estimated the odds ratio (OR) measuring the association between model variables and cow bunching. The OR ranges from 0 to infinity with an OR equal to 1 being no association, values less than 1 indicative of a protective effect, greater than 1 indicative of a risk factor. A statistically significant association can be inferred when the 95% confidence intervals of the OR does not include 1.

*Stable fly count per cow leg*. Eq 1 summarizes the GLMM used to model cow bunching observed at the pen level by number of stable flies observed on cow legs.

$$Logit\left[P({Bunching}_{mijkl})\right]=\beta_{0}+\beta X+t_{m}+u_{mi}+v_{mij}+w_{k}+z_{kl}$$ Eq [1]

**Fig 2:**
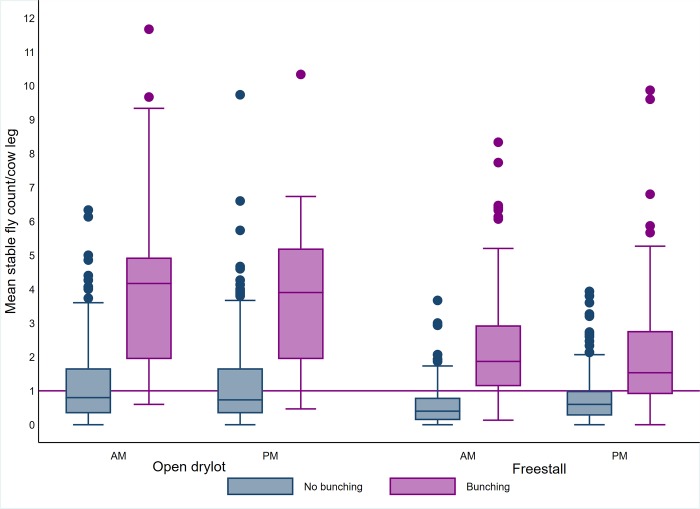
Schematic presentation of a longitudinal study of cow bunching using cow leg fly count at the pen level. Pens were nested within dairies, which were nested within observers. For example, nesting implies that pen 1 in dairy 1 differs than pen 1 in other dairies. Pens were also crossed by time of day which were nested within week. For example, crossing implies that the AM bunching observations completed at the same morning on all the pens in all the dairies.

Five random effect variables were included in the model including observer, dairy, pen, week, and time of day of fly counts on cow legs (AM or PM). Pens were nested within the dairy and am/pm counts were nested within week. However, pens were crossed with week and time of day of stable fly counts (Fig 2). The dairies were nested within observers; *m* = 2 [16]. In dairy *i*, *i* = {1,2,3,4,5,7,8,9,10}, were pens *j*, where *j* for *i* = 10,*j* = {1,2,3,4} while for the remaining 9 dairies *j* = {1,2,3}. During week *k*,*k* = {1,2,..,13}, stable fly counts were recorded at the *l* time of day, where *l* = {*AM*,*PM*}. Where the random effects for observer, dairy, pen, week and time of day were *t*_*m*_,*u*_*i*_,*v*_*j*_,*w*_*k*_ *and z*_*l*_, respectively.

**Fig 3:**
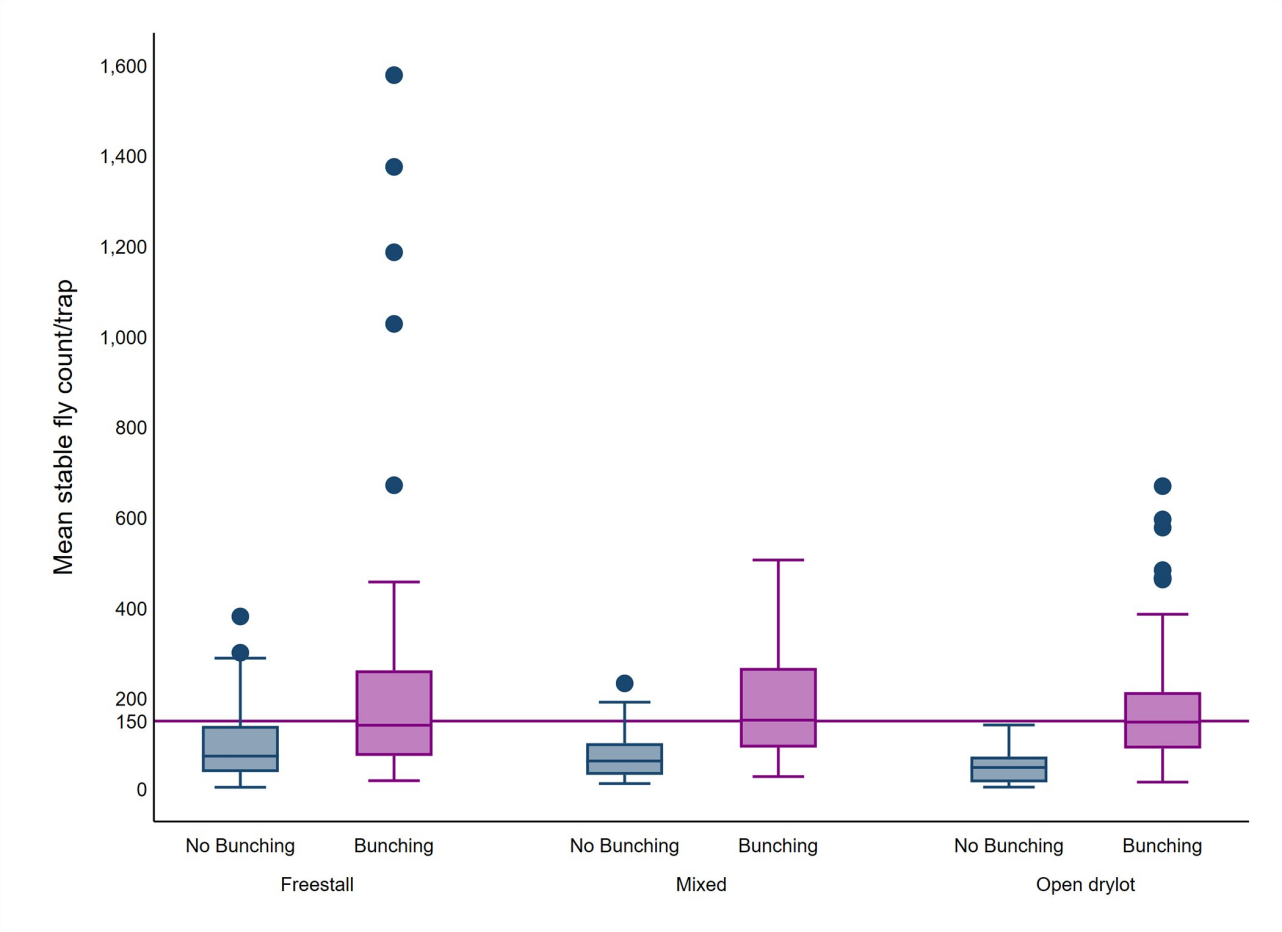
Boxplot of average stable fly count on cow leg by bunching, time of day and pen design. The upper and lower horizontal lines of the box represent the 25^th^ and 75^th^ percentiles respectively, the horizontal midline represents the median, the upper and lower horizontal whiskers lines are the upper and lower limits and the dots are the outliers.

**Fig 4:**
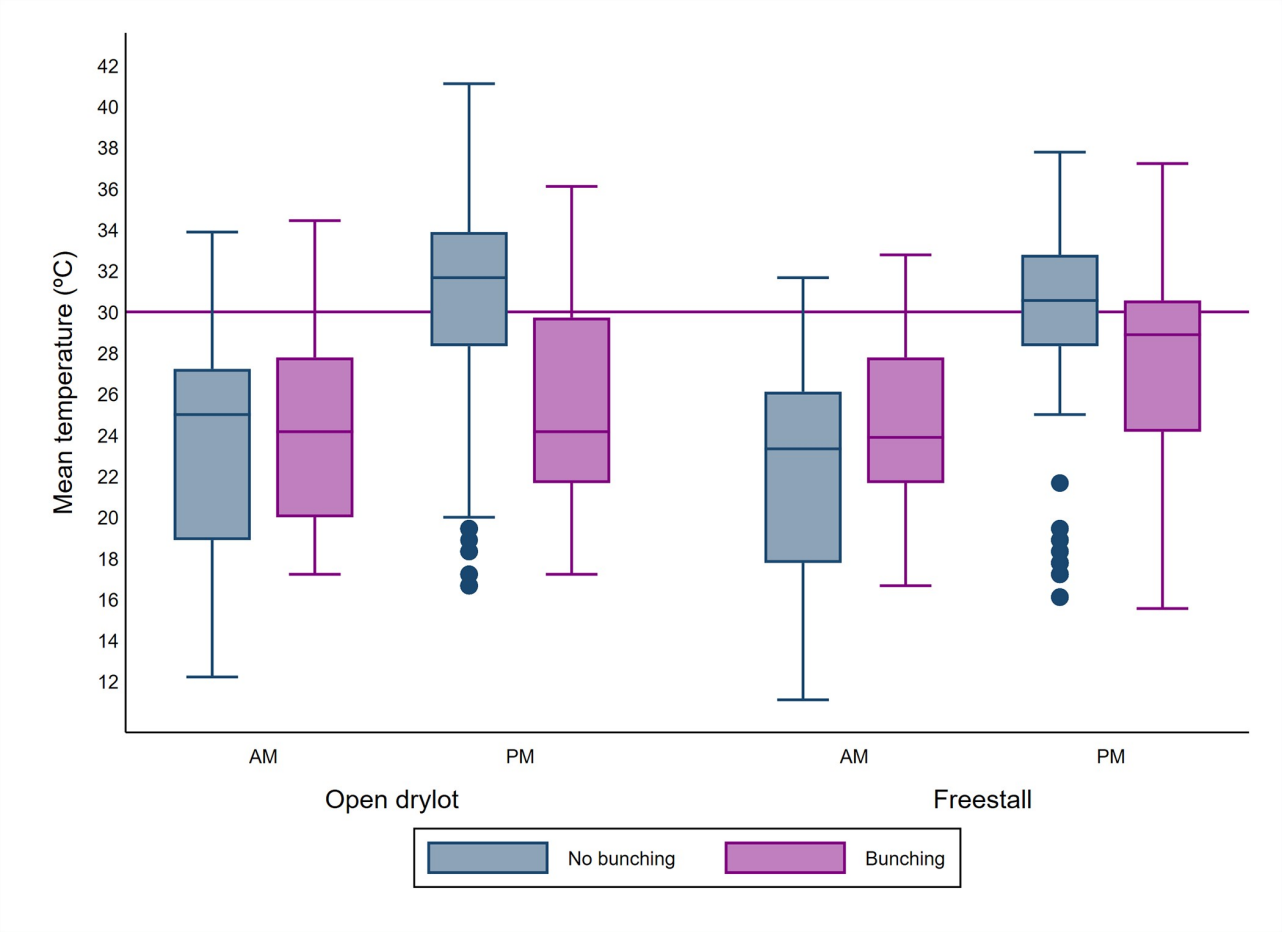
Boxplot of average trap count, bunching and farm design. The upper and lower horizontal lines of the box represent the 25^th^ and 75^th^ percentiles respectively, the horizontal midline represents the median, the upper and lower horizontal whiskers lines are the upper and lower limits and the dots are the outliers.

**Fig 5:**
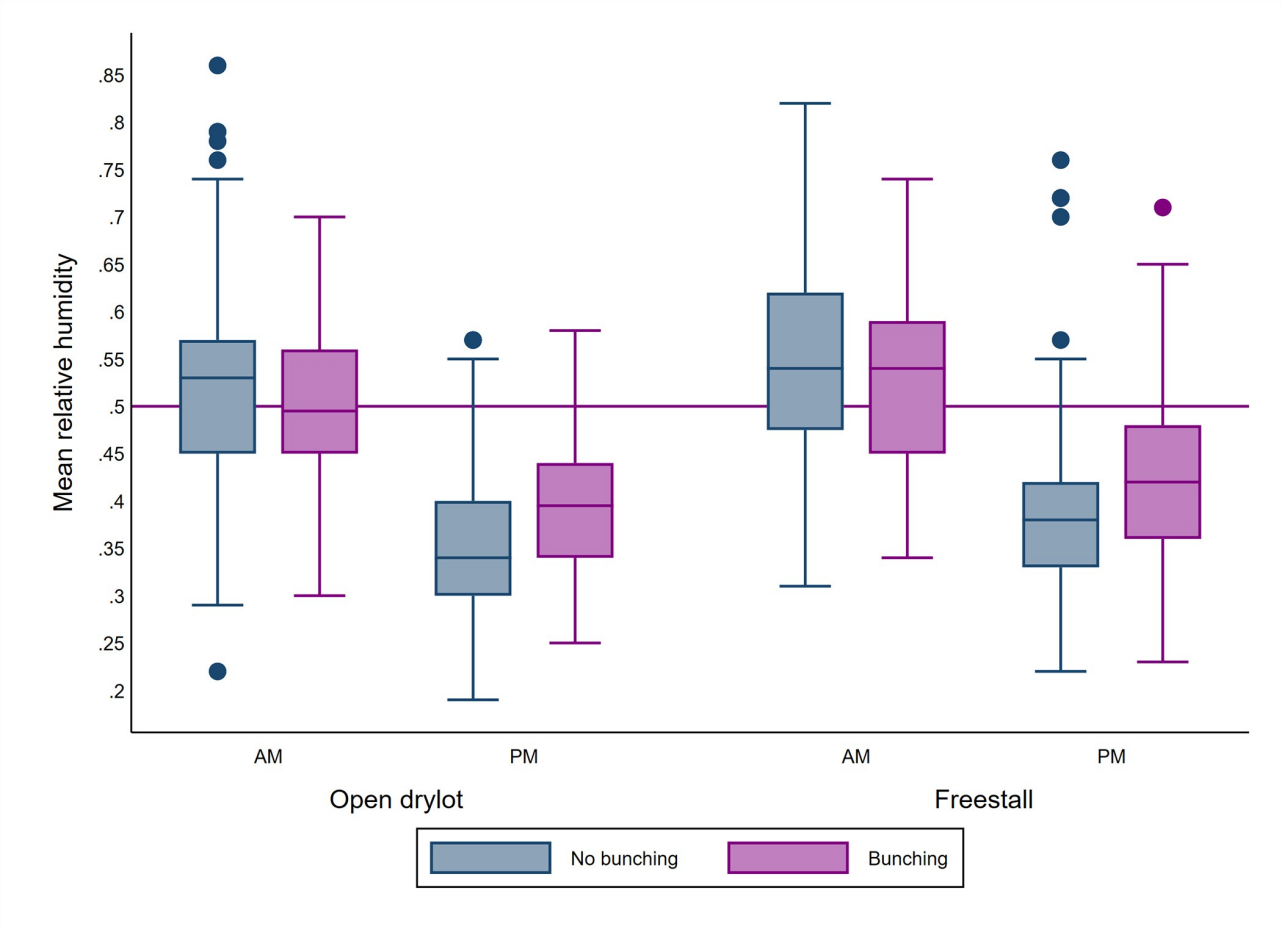
Boxplot of mean ambient temperature(°C) during AM and PM counts and bunching according to the pen design. The upper and lower horizontal lines of the box represent the 25^th^ and 75^th^ percentiles respectively, the horizontal midline represents the median, the upper and lower horizontal whiskers lines are the upper and lower limits and the dots are the outliers.

**Fig 6:**
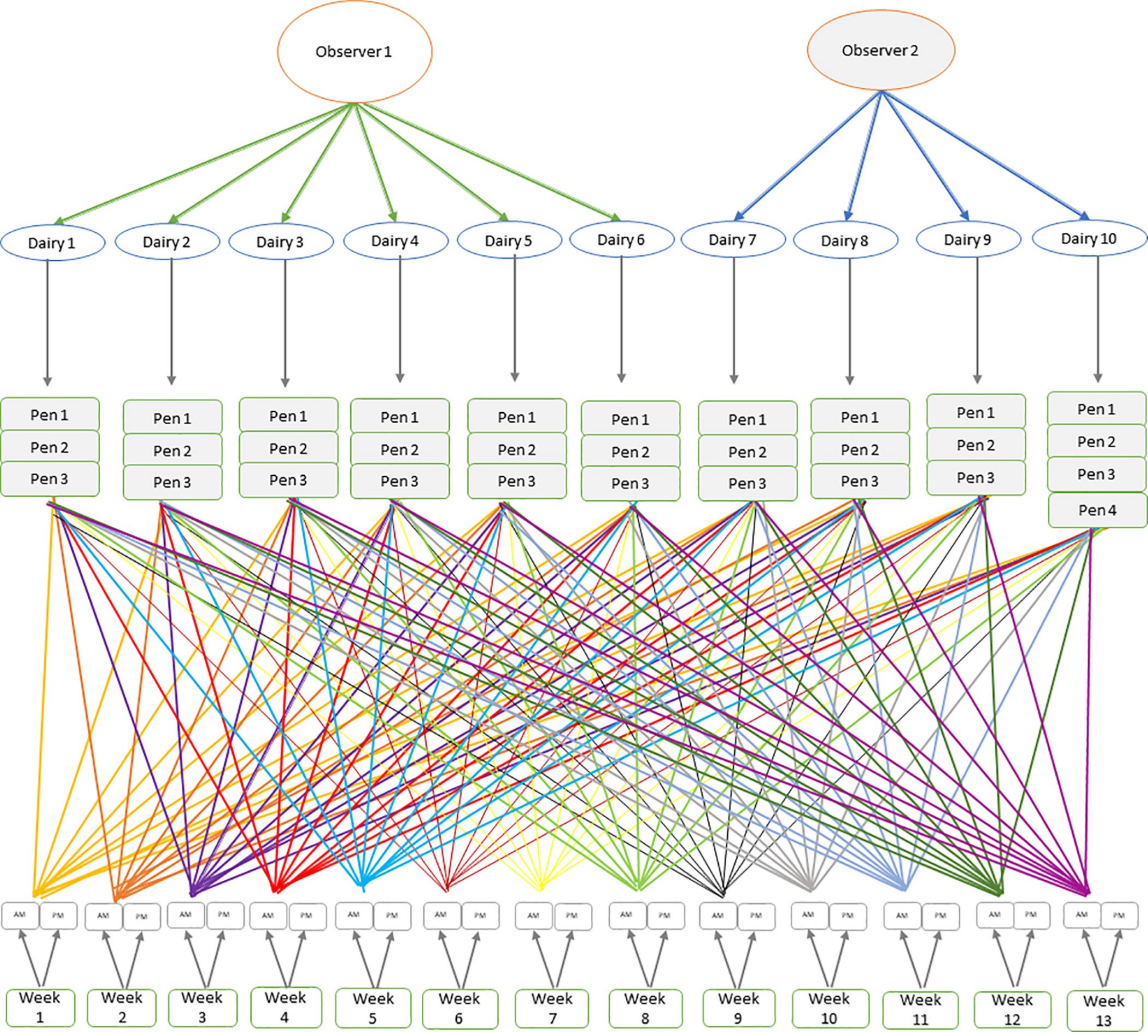
Boxplot of mean relative humidity during AM and PM counts and bunching according to the pen design. The upper and lower horizontal lines of the box represent the 25^th^ and 75^th^ percentiles respectively, the horizontal midline represents the median, the upper and lower horizontal whiskers lines are the upper and lower limits and the dots are the outliers.

In addition to the intercept (*β*_0_), the fixed effect variables (*βX*) were related to 2 main groups, dairy level factors and pen level factors. The dairy and pen level explanatory variables were introduced to the model. However, the average leg count observed during each of the am and pm fly count visits were used. Similarly, ambient temperature and relative humidity observed during each am and pm fly count visits were used. A preliminary analysis was used to select the variables to be included in the model. All the variables were included with the random effect variables as univariate models with 5% level of significance in all tests. Continuous variables including the average leg count (Fig 3), average trap count (Fig 4), ambient temperature (Fig 5), relative humidity (Fig 6), weeks and times milked were transformed into categorical variables. Categories of the variables were determined using exploratory analyses including descriptive statistics to identify the mean and median, quartiles and box plots. For each variable, the different categorization options were contrasted using a univariate model and the option that produced the lowest Akaike Information Criterion (AIC) was selected [24].

Non-significant variables in the univariate analysis were grouped based on the biological plausibility of their association with stable flies: fence line manure, manure mounds and wet spots inside the pen were grouped into one variable called decomposed organic matter while feed refusal and the condition of the feed curb were grouped as one variable called decomposed vegetation. A manual backward model building process was employed based on significant univariates followed by reentry of variables that dropped in earlier steps. During the variable selection and model building, confounding by known confounders was assessed using the method of change in estimates and two-way interactions for potential effect modifiers were tested using significance testing. The AIC estimate was used to select between competing models with lower values denoting better model goodness of fit [24].

*Trap counts*. Eq 2 summarizes the GLMM used to model cow bunching observed at the pen level on all 20 study dairies where fly counts observed on 5 traps on each dairy were recorded.

Cow bunching (*y*_*mijkl*_) as an outcome was observed at the pen level was analyzed using a multilevel effect mixed model as in Eq (2).

$$Logit\left[P\left(Bunching_{ijkm}\right)\right]=\beta_{0}+\beta X+u_{i}+v_{ij}+w_{k}+t_{m}$$ Eq [2]

**Fig 7:**
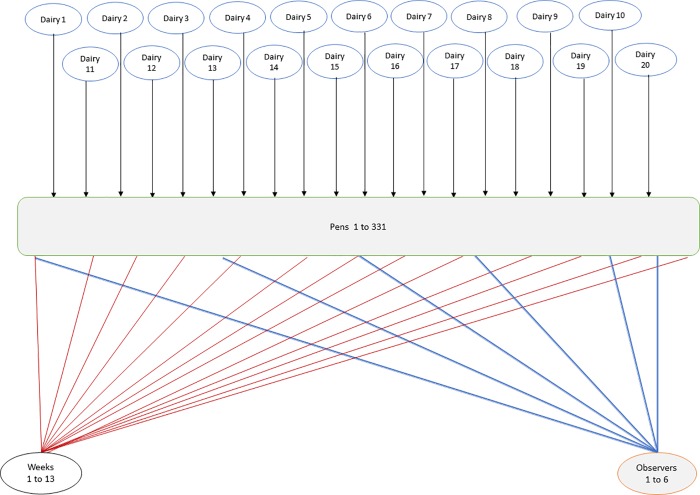
Schematic presentation of a longitudinal study of cow bunching at the pen level using trap fly count/pen. Pens were nested in dairies and crossed by observers and weeks. For example, nesting implies that pen 1 in dairy 1 differs than pen 1 in other dairies. Crossing implies that the weeks of bunching observations, for example, were the same weeks on all the pens.

Four random effect variables were included in the model including, dairy, pen, week, and observer. Pens were nested within the dairy and were crossed with week and observer of stable fly counts on fly traps (Fig 7). In dairy *i*, *i* = {1,2,……,20}, were pens *j*, where *j* = {1,……,331}, for the entire study period, week *k*,*k* = {1,2,..,13}, and observer *m* stable fly count on the traps; *m* = {1, …, 6}. Where the random effects for dairy, pen, week, and observer were *u*_*i*_,*v*_*j*_,*w*_*k*_ *and t*_*m*_, respectively.

The fixed effect variables were related to 2 main groups, dairy level factors and pen level factors. The dairy and pen level factors detailed above were introduced to the model. However, the average stable fly count variable was specified as the closest fly trap count (if the pen is equally located between 2 traps the mean of both traps was related to the pen). Similarly, the average of weekly ambient temperature and relative humidity were used as model variables. A preliminary analysis was used to select the variables to be included in the model. All the variables were included with the random effect variables as univariate models with 5% level of significance in all tests. Continuous variables including the weekly stable fly count on the trap closest to the pen, the mean weekly ambient temperature, and relative humidity, weeks and times milked were transformed into categorical variables. Categories of the variables were determined using exploratory analyses including descriptive statistics to identify the mean and median, quartiles and box plots. For each variable, the different categorization options were contrasted using a univariate model and the option that produced the lowest AIC was selected [24].

Non-significant variables in the univariate analysis were grouped based on biological plausibility of their association with stable flies as in the leg count model building described above. A manual backward model building process was employed based on significant univariates followed by reentry of variables that dropped in earlier steps. During variable selection and model building, confounding by known confounders was assessed using the method of change in estimates and two-way interactions for potential effect modifiers were tested using significance testing. The AIC estimate was used to select between competing models with lower values denoting better model goodness of fit [24].

#### Modeling Cow Bunching at the Dairy Level

Eq 3 summarizes the GLMM used to model cow bunching observed weekly on all 20 study dairies and recorded primarily during trap fly count sessions. The OR were interpreted as described above. However, bunching was also verified during the cow leg fly count sessions and by owner/ herd manager reported bunching episodes on their dairies.

$$Logit\left[P({Bunching}_{ikm})\right]=\beta_{0}+\beta X+u_{i}+w_{k}+t_{m}$$ Eq [3]

**Fig 8:**
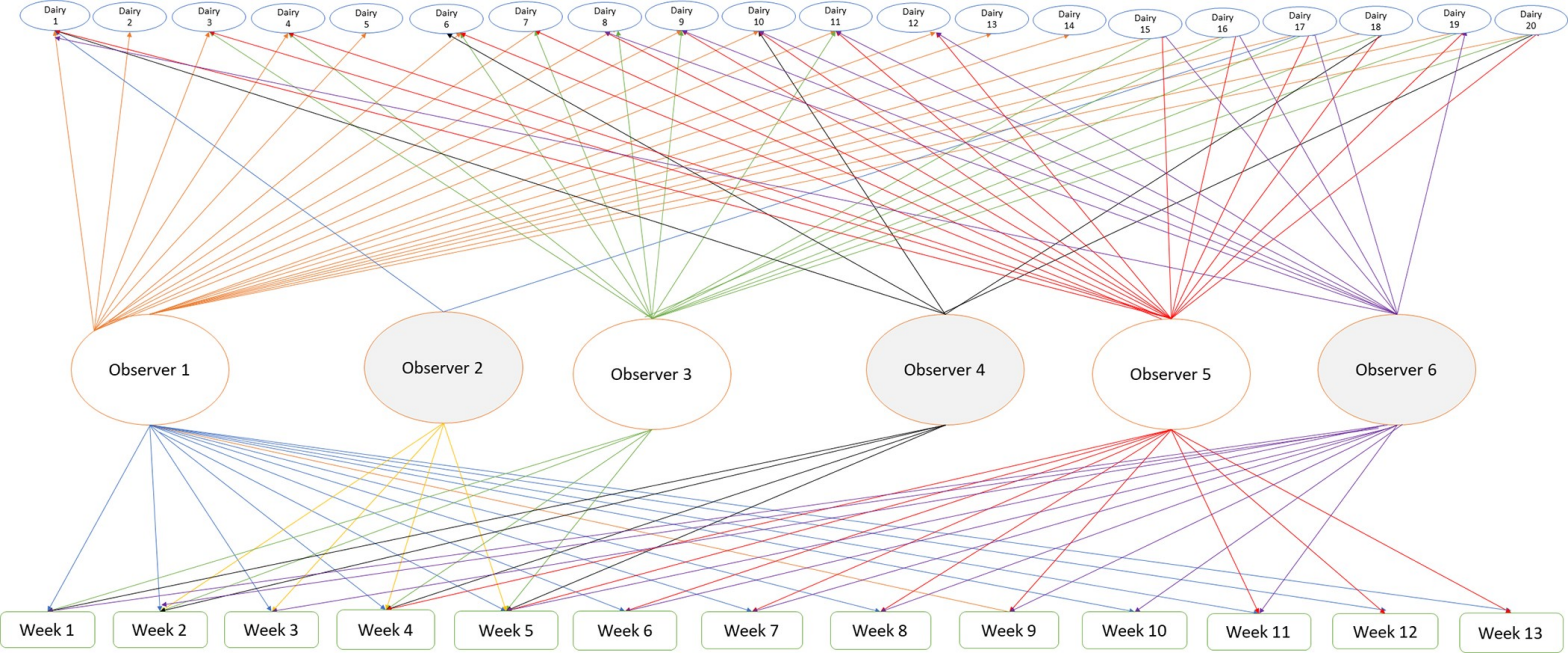
Schematic presentation of a longitudinal study of cow bunching at the dairy level using the average trap fly count. Dairies were crossed by observers and weeks. Crossing implies that the weeks of bunching observations, for example, were the same weeks on all the pens.

Three random effect variables were included in the model including dairy, week, and the observer recording bunching on the dairy during fly trap counting days. Dairies were crossed with week and the observer of stable fly count on the fly traps (Fig 8). Dairy *i*, *i* = {1,2,……,20}, *k* for week, *k* = {1,2,..,13} and *m* for observer, m = {1,2,3,4,5,6}. Where the random effects for dairy, week, and observer were *u*_*i*_,*w*_*k*_ *and t*_*m*_ respectively

The fixed effect variables were related to the dairy explanatory variables were introduced to the model. However, the average weekly stable fly count on the 5 traps, the mean weekly ambient temperature and relative humidity were used. A preliminary analysis was used to select the variables to be included in the model. All the variables were included with the random effect variables as univariate models with 5% level of significance in all tests. Continuous variables including the mean weekly stable fly count on the 5 traps (Fig 7), the mean weekly ambient temperature, and relative humidity, weeks and times milked were transformed into categorical variables. Categories of the variables were determined using exploratory analyses including descriptive statistics to identify the mean and median, quartiles and finally box plots. For each variable, the different categorization options were contrasted using a univariate model and the option that produced the lowest AIC was selected [24].

All significant variables in the univariate model were fitted jointly in a multivariate model, and variables that were no longer statistically significant were excluded from the final model. A manual backward model building process was employed based on significant univariates followed by reentry of variables that dropped in earlier steps. During the variable selection and model building, confounding by known confounders was assessed using the method of change in estimates and two-way interactions for potential effect modifiers were tested using significance testing. The AIC estimate was used to select between competing models with lower values denoting better model goodness of fit[24]. The computer software used for all statistical analyses was Stata 15.1®.

## Results

### Survey responses related to bunching and fly control

Bunching was reported for all but one of the study herds during April to July of the year prior to commencement of the study. Regarding the possible cause of bunching in these herds, 12 herd mangers reported that bunching was related to flies, 6 specified biting flies and 2 reported it as a behavioral issue. Of the 20 study herds, 13 herd managers reported decrease in milk production, 9 reported decrease in dry matter intake and 4 noticed increase in cases of lameness during bunching. Nineteen of the study dairies had a fly control program, 10 hired a company for the fly control (4 relied solely on the hired company while 6 used additional methods). Seventeen dairies used products to control stable flies including predatory wasps, insecticidal sprays, and/or addition of larval inhibitor to lactating rations Table 1.

**Table 1:** Data description for the study dairies.

**Variable**	**Unit**	**Frequency**	**Estimate**	**Std. Err.**	**95% Confidence limits**	
					**Lower**	**Upper**
**Farm design**		20				
Freestall	%	10	50	0.112	27	73
Open Dry lot	%	5	25	0.097	8.7	49
Mixed	%	5	25	0.097	8.7	49
Breed		20				
Holstein	%	13	65	0.107	41	85
Jersey	%	4	20	0.09	6	44
Both	%	3	15	0.080	3	38
**Animals in the facility**		20				
calves	%	4	20	0.110	19	64
Heifers	%	20	100	0.000	83	100
Springers	%	20	100	0.000	83	100
Rolling herd average	Kg/cow/year	20	26285.95	937.902	24322.90	28249
Number of Lactating cows	Number	20	2466.25	234.761	1974.89	2957.61
**Calves management**						
Housing						
Raised individually	%	8	1.00	0.000	63	100
Wooden hutches	%	8	0.88	0.117	47	100
Plastic hutches	%	8	0.13	0.117	0	53
Hutches raised	%	8	0.88	0.117	47	100
Flush under hutches	%	8	0.63	0.171	24	91
Flush clean water	%	5	0.40	0.219	5	85
Removal calf refusal	%	7	0.71	0.171	29	96
**Manure management**						
Freestall cleaning	%	15	1.00	0.000	78	100
Removal of fecal pats	%	15	0.67	0.126	40	94
Freestall bedding	%	15	1.00	0.000	78	100
Freestall raking	Times/month	15	27.40	3.156	20.63	34.17
Freestall refill/month	Times/month	15	3.73	0.643	2.35	5.11
Dirt corral rake/month	Times/month	19	11.05	2.506	5.79	16.32
Dirt corral scrape/year	Times/year	19	21.42	5.137	10.63	32.21
Dirt corral bedding/year	Times/year	19	19	30.334	5.39	7.67
Feed lane flush/day	Times/day	20	3.15	0.264	2.60	3.70
Feed lane scrape/month	Times/month	20	13.6	3.226	6.85	20.35
Feed lane scrape	%	20	0.55	0.111	32	77
Cleaning feed curb LC/year	Times/year	20	15.10	4.003	6.72	23.48
Cleaning fenceline manure	%	20	0.45	0.111	23	68
Solid manure storage	%	20	1.00	0.000	83	100
Covered manure piles	%	20	0.2	0.089	6	44
Manure removal (2017)	%	20	0.3	0.102	12	54
Solid manure removal/year	Times/year	20	2.88	0.473	1.89	3.86
Number of lagoons	Number	20	3.60	0.380	2.81	4.39
**Cont’d Variable**	**Unit**	**Frequency**	**Estimate**	**Std. Err.**	**95% Confidence limits**	
					**Lower**	**Upper**
Acres/wastewater around dairy	Number	18	510.33	80.009	341.53	679.14
Wastewater/crops around dairy	Number	20	0.95	0.049	0.75	2.00
**Feed management**						
Feeding by-products	%	20	100	0.000	83	100
Wet distiller grains	%	20	5	0.112	27	73
Fruits by-products	%	20	25	0.097	8.7	49.1
Vegetables by-products	%	20	20	0.089	6	0.44
Almond hulls	%	20	90	0.067	68	0.99
Cotton seed	%	20	100	0.000	83	100
Adding fluids to ration						
Water	%	20	35	0.110	15	59
Molasses	%	20	25	0.097	9	49
Whey	%	20	30	0.102	12	54
Times fed LC/day	Times/day	20	1.88	0.114	16.4	21.1
Times push up feed LC/day						
= <6	Times/day	20	0.4	0.110	0.191	0.64
>6	Times/day	20	0.6	0.110	0.36	0.81
**Cow cooling**						
Soakers	%	20	1	0	83	100
Fans_	%	20	65	0.107	41	85
**History of bunching**	%					
Bunching previous years	%	20	95	0.049	75	100
Affect milk production	%	19	68	0.107	43	87
Affect dry matter intake	%	19	47	0.115	24	71
Increase lameness	%	18	22	0.098	6	48
Bunching possible causes						
Flies	%	19	63	0.111	38	84
Behavior	%	19	11	0.070	1	33
Biting flies	%	19	32	0.107	0.13	57
**Fly control**						
Fly control program	%	20	95	0.049	75	100
Hire a company	%	20	50	0.112	27	73
Other programs	%	20	75	0.097	51	91
Stable fly product	%	20	85	0.080	62	97

### Descriptive statistics

#### Herd demographics

The study herds were Holstein (16 herds), Jersey (2 herds), or mixed Jersey and Holstein breeds (2 herds). The average number of lactating cows per herd was 2,425 (SE±1050) with a minimum of 725 cows and maximum of 5,000 cows. The mean rolling herd average milk production of all study herds was 11,948 kg, SE±1,906 (26,286 lb), with a minimum of 8,627 kgs (18,980 lb) and maximum of 15,264 kg (33,580 lb). The enrolled herds were milked either twice a day (12 herds) or three times a day (7 herds) and only one herd had its lactating cows milked either twice and three time per day depending on their productivity and days in milk.

The 20 study herds (lactating and dry cows) were housed in either freestall with or without exercise area (8 herds), open dry lot (5 herds) or mixed design with both freestall and open dry lot pens (7 herds). Dairies with a mixed design were either freestall with exercise area and open dry lot (4 herds), freestall without exercise area and open dry lot (one herd); or dairies with the 3 pen varieties freestall with exercise area, freestall without exercise area and open dry lot (2 herds). All but one of the study herds housed their dry and close-up (3 weeks before calving) cows on the same property. The remaining herd’s springers (uniparous pregnant heifers 3 weeks within due date), dry and close-up female cattle were housed on another study herd belonging to the same owner. Close-up cows were housed in open dry lot pens (16 herds), bedded pack (1 herd), open dry lot and bedded pack (1 herd), and freestall pens without exercise area and bedded pack (1 herd). Growing and replacement heifers in all the study herds were raised on the same premises while, only 8 herds raised their calves on the same facility, Table 1.

#### Manure management

The 15 study dairies with freestall pens were either bedded with dried manure (10 dairies) or recycled sand (5 dairies). Fecal pat removal from the freestall beds was performed on 10 of the 15 freestall dairies. The freestalls were raked on average 27.4 times per month (SE±3.156) and refilled 3.73 (SE ± 0.643) times per month. On dairies with dirt corrals, the mean rate for raking was 11.05 times per month (SE ± 2.506); while the annual rates for scraping and bedding were 21.42 (SE ± 5.137), and 19 (SE ± 30.33), respectively. Fence line manure was cleaned on 9 of the 20 study dairies. All study dairies stored the solid manure on the same site either in covered by plastic tarps (4 herds) or uncovered manure mounds (16 herds). The median number of recycled flush water lagoons on the study dairies was 3 and ranged from 1 to 7. All but one of the study dairies applied recycled wastewater to farmland surrounding their premises with a mean of 510 Acres/dairy (SE± 80). The alleyways were flushed with recycled lagoon water at a mean rate of 3.15 times per day (SE ± 0.264), however, 11 of freestall dairies used scrapers for the alleyway at a rate of twice per day (one dairy), once per day (8 dairies), once every 2 days (one dairy) and once every 2 weeks (one dairy). Feed curb was cleaned at a mean rate of 15.1 times per year (SE ± 4.003) on 19 of the 20 dairies, the remaining dairy’s management did not clean the feed curb, Table 1.

#### Feed bunk management

Each of the 20 study dairies had a commodity barn and ensiled feed on site. Silage storage was either drive over piles (5 dairies), steep sided piles (6 dairies), silage bags (3 dairies), or a combination of drive over piles and concrete sided piles (one dairy), steep sided piles and silage bags (one dairy), drive over piles and silage bags (one dairy) or steep-sided piles and drive over piles (3 dairies). By-products were fed on all study dairies, 10 herds fed wet distiller grain, 4 fed vegetable by-products, 5 fed fruit by-products, 18 fed almond hulls, and 20 fed cotton seed. In addition, water was added to lactating rations (7 herds), dry cow ration (2 herds), and close-up ration (4 herds). Whey was added to the lactating ration (6 herds), dry cows ration (one herd), and close-up ration (2 herds). Molasses was added to lactating ration (5 herds), dry cows ration (one herd) and close-up ration (2 herds). Lactating cows were fed either once (4 herds), twice (14 herds) or three times a day (one herd). Only one herd fed the low milk production cows twice/day and high milk production cows 3 times/day. Feed was pushed up in the feed bunk either ≤ 6 times/day (8 herds) or > 6 times/day (12 herds), Table 1.

#### Heat stress abatement

Cooling of the 20 study herds was done using either soakers, misters and/or fans. All the study dairies had soakers for cooling lactating cows, in addition, 13 herds also had fans. For cooling dry cows, 9 dairies had soakers and only one also had fans. For cooling close-up cows, 2 herds had misters and 12 had soakers, 9 of the latter also had fans, Table 1.

### Cow bunching

#### Pen-level cow bunching

*Stable fly count per cow leg*. Stable fly counts on cow legs were recorded on 10 dairies. Descriptive statistics on those 10 dairies are presented in the S3 Appendix. Results of the model predicting cow bunching at the pen level as explained by fly counts on cow legs are presented in Table 2. Variability (SE) in the bunching attributable to observer, dairy, pen, week and time of day were 0.39 (0.620), 0.84 (0.943), 1.56 (0.976), 0.29 (0.512), and 1.14 (5.97) respectively.

**Table 2:** Final generalized linear mixed model with logit link predicting cow bunching at the pen level using Stable fly counts on cow legs on 10 California dairies.

Factor	Level	Odds Ratio ^a^	Standard error	P-Value	95% Confidence limits	
					Lower	Upper
Average leg count	≤ 1	Reference				
	>1	22.94	9.749	< 0.01	9.97	52.76
Pen design	Open dry lot	Reference				
	Freestall	12.09	9.914	< 0.01	2.42	60.33
Ambient temperature	> 30 C° (95 F)	Reference				
	≤ 30 C° (95F)	2.09	0.758	0.04	1.03	4.26
Relative humidity	≤ 50%	Reference				
	> 50%	0.45	0.152	0.02	0.23	0.87
Weeks	(9–13)^b^	Reference				
	(1–8)^c^	8.29	3.239	< 0.01	3.85	17.83
Pen surroundings	Others	Reference				
	Crops	5.22	3.529	0.01	1.39	19.63

^a^ The ratio of odds of cow bunching at the pen level in the presence of the factor to that in the absence of the factor.

^b^26^th^ June 2017 to 31^st^ July2017

^c^1^st^ May 2017to 25^th^ June 2017

In addition to the average leg count, other risk factors (higher odds) for cow bunching in the counted pens were freestall pen design (versus open dry lot), ≤ 30°C ambient temperature (versus > 30°C), months of May and June (versus July), and presence of wheat/corn or alfalfa crops on one or more sides of the pen (versus no crops, e.g. roads, other dairy pens, trees, etc.). Relative humidity of > 50% (versus ≤50%) was the only protective factor against bunching.

*Trap counts*. The results of the model predicting cow bunching at the pen level as explained by fly counts on traps are presented in Table 3. Variability (SE) in the bunching attributable to dairy, pen, week and observer were 1.29 (0.491), 0.97 (0.210), and 0.18 (0.168) respectively.

**Table 3:** Final generalized linear mixed model with logit link predicting cow bunching at the pen level (331 pens) using Stable fly counts on traps placed on 20 California dairies.

Variable	Level	Odds Ratio	Standard error	P-Value	95% Confidence limits	
					Lower	Upper
Stable fly counts on trap	≤50	Reference				
	>50	2.21	0.319	< 0.01	1.67	2.93
Weeks	(9–13) *	Reference				
	(1–8) **	8.82	1.335	< 0.01	6.55	11.86
Pen design	ODL&BP^Δ^	Reference				
	FSE^ΔΔ^	3.47	0.972	< 0.01	2.01	6.01
	FSnoE^ΔΔΔ^	2.89	1.346	0.02	1.16	7.20
Production status	Close-up	Reference				
	Lactating	3.86	1.726	< 0.01	1.61	9.27
	Dry	4.29	1.755	< 0.01	1.93	9.57
Pen surroundings (crops)	No	Reference				
	Yes	2.11	0.551	< 0.01	1.76	38.24
Pen surroundings (dairy)	<4 sides	Reference				
	= 4 sides	0.41	0.099	< 0.01	0.25	0.66
Pen surroundings (main road)	No	Reference				
	Yes	0.46	0.139	0.01	0.26	0.83
Cont’d Variable	Level	Odds Ratio	Standard error	P-Value	95% Confidence limits	
					Lower	Upper
Molasses added to ration	No	Reference				
	Yes	2.20	0.842	0.04	1.04	4.66

*26^th^ June 2017 to 31^st^ July2017

** 1^st^ May 2017to 25^th^ June 2107

^Δ^ Open dry lot or bedded pack

^ΔΔ^ Freestall with exercise area

^Δ Δ Δ^ Freestall without Exercise area

Risk factors for cow bunching at the pen level included trap counts >50 fly/trap/week, free stall pen design with and without exercise area, the months of May and June, lactating and dry cow production, presence of wheat/corn or alfalfa crops on one or more sides of the pen, and addition of molasses to the ration. However, odds of bunching decreased if the pen had a major road on one or more sides, was surrounded on all 4 sides by other pens, or was located adjacent to any structures on the dairy such as a feed commodity area, parlor, office or work shed etc..

#### Dairy level cow bunching

The results of the model for cow bunching at the dairy level using fly counts on traps are presented in Table 4. Variability (SE) in the bunching attributable to dairy, week and observer were 1.01 (0.734), 1.09 (0.843), and 0.06 (0.340) respectively.

**Table 4:** Final generalized linear mixed model with logit link predicting cow bunching at the dairy level using trap Stable fly counts on 20 California dairies.

Variable	Level	Odds Ratio	Standard error	P-Value	95% Confidence limits	
					Lower	Upper
Trap stable fly count	< 150	Reference				
	≥ 150	7.72	5.308	<0.01	2.00	29.70
Mean ambient temperature	C°	0.72	0.101	0.02	0.55	0.95
Weeks	(9–13) *	Reference				
	(1–8) **	16.27	16.008	<0.01	2.36	111.94
Feeding wet distiller grains	No	Reference				
	Yes	4.41	3.219	0.04	1.06	18.43
Cleaning fence line manure	No	Reference				
	Yes	0.013	0.013	<0.01	0.00	0.10
Crops surrounding farms	≤ 2 sides	Reference				
	≥3 sides	5.30	4.291	0.03	1.08	25.91

*26^th^ June 2017 to 31^st^ July2017

** 1^st^ May 2017to 25^th^ June 2107

Risk factors for cow bunching at the dairy level included trap count ≥150 fly/trap/week, the months of May and June (versus July), feeding wet distiller grains, and presence of wheat/corn or alfalfa crops on one or more sides of the pen. Higher mean ambient temperatures and cleaning the fence line manure were protective.

## Discussion

Findings from this observational study showed that bunching behavior was positively associated with average trap count ≥150 flies/trap, stable fly season (May through June, compared to July), feeding wet distiller grain, presence of crops around the dairy and accumulation of fence line manure. In contrast, high temperature and relative humidity were negatively associated with bunching. At the pen level, bunching was positively associated with average leg count > 1 stable fly/leg/cow, and stable fly trap counts closest to a pen with >50 flies/trap. Other pen-level factors associated with bunching included freestall pen design (compared to open dry lot), stable fly season (May through June, compared to July), production status (dry or lactating compared to close-up cows), presence of crops surrounding the pen, addition of molasses to the ration, and ambient temperatures (≤30°C) and relative humidity < 50%.

The number of stable flies on fly traps reflects the abundance and activity of stable flies in the near vicinity [25]. Increasing stable fly biting activity around cows initiates a protective fly repelling behavior by cows inside the pen. Such behavior may be individual in the form of leg stomping, tail switching, skin tingling and head throwing, or bunching as a group behavior. Animals in the group are protected by their proximity to other individuals and by being in the center of the group, hence creating a dilution effect which reduces the fly attack [12, 13, 17, 18]. Dairies with a mean weekly stable fly count of ≥150 flies/trap had higher odds of bunching compared to dairies with lower counts. For traps closest to an individual pen, a lower trap count of >50 flies/trap(s) was significantly associated with bunching in that pen suggesting that proximity of the trap to the pen provided greater sensitivity as a predictor of bunching. Even greater sensitivity is noted in the association of stable fly counts on the legs of cattle within a single pen with average leg count > 1 stable fly on cow legs associated with bunching in the pen. While this level of stable fly biting activity might seem too low to impact animal behavior, the bites of these flies are quite painful and previous studies have shown measurable negative impacts to cattle (such as reduced weight gain) when stable fly biting activity was as low as 2.5 flies/leg[26].

May and June (study weeks 1 to 8) were associated with elevated risk for cow bunching compared to later weeks. The study week effect was consistent between models, specifically, among dairies, between pens and using both fly counts on traps and cow legs. A difference in risk of cow bunching by week may be attributable to the abundance and activity of stable flies over the months evaluated in the current study. Mullens, *et al*. [18, 20] reported that the maximum seasonal activity of stable flies on California dairies occurs from May through June, with stable fly activity decreasing in July as daytime temperature begins to peak [18, 20]. In fact, stable fly activity is greatly impacted by environmental conditions, with maximum biting activity occurring at 24–30°C [18] and at low humidity (7% RH) with high humidity reducing biting activity [27]. Reproduction is also impacted by environmental conditions with maximum fecundity occurring at 25–30°C [28]. Climatic conditions such as ambient temperature and relative humidity were associated with cow bunching in the current study. Specifically, temperatures > 30° C and relative humidity > 50% recorded at time of stable fly counts on cow legs were associated with lower odds of cow bunching, perhaps due to the negative impacts of these environmental conditions on stable fly survival and activity [25, 29].

Feed bunk management, feeding wet distiller grains and addition of molasses to the ration are risk factors for bunching. Dairies that fed wet distiller grains had higher odds of bunching than dairies that did not. At the pen level, pens with molasses added in their ration had higher odds of bunching than pens without molasses in their ration. These observational findings point to the potential indirect role of feed ingredients on bunching, perhaps through increased stable fly survival as a result of fly feeding on sucrose or other sugars available in these feed ingredients to supplement bloodmeals from the nearby cattle [12, 30]. In addition, fermented products such as wet distiller’s grain may provide a good habitat for the oviposition and development of stable flies.

Cleaning of fence line manure was protective against bunching among dairies. Manure build up under the fence line provides a good habitat for stable fly development [31] as the outer layer of the manure can make a protective crust against sunlight and provide the optimum environmental conditions for the eggs, larvae and pupae of stable flies [32, 33].

Crops including wheat/corn or alfalfa grown in fields surrounding the study dairies and their pens were risk factors for cow bunching. Dairies surrounding by wheat/corn or alfalfa fields on 3–4 sides of the dairy had higher odds of cow bunching relative to dairies with fewer surrounding crop fields. While pens with one or two sides surrounded by crops had higher odds of cow bunching compared to pens without bordering crop fields. Wheat/corn and alfalfa may provide a good habitat for the adult stable flies for resting and mating behavior in addition to their blooms being a source of sugars [12, 28]. Cows in pens surrounded on all sides by other pens or bordered by a main road were protected from bunching, suggesting a dilution effect as stable flies must first pass through surrounding pens containing cattle to reach protected animals or perhaps due to the greater distance between cattle and crop fields that serve as stable fly resting and sugar feeding sites[30]

Freestall pens with or without exercise areas were at higher odds of bunching in comparison to open dry lot and bedded pack pens. Freestall pens have numerous metal structures which may provide good resting sites for stable flies to bask in the sun to warm up before biting cattle in early morning [12, 33, 34] or to find refuge in the shade during hot afternoons. Freestall designs are therefore well suited to keeping adult stable flies near their cattle hosts. The magnitude of the association between pen design and bunching was greatest when fly activity was determined using leg counts rather than trap counts. Leg counts are a direct measure of fly biting activity, while fly trap counts represent a week’s worth of general fly activity.

Production status was also associated with bunching. Lactating cows and dry cows had higher odds of bunching in comparison to close-up cows, perhaps due to differences in animal management including pen design, as cows in most of the close-up pens were housed on bedded packs with good cow cooling systems including soakers and fans. In contrast, lactating cows are exposed to the stress of lactation and most of the dry cows are housed in open dry lot pens with or without shade. Furthermore, differences in rations fed to the different production states may be associated with bunching including times fed and ration ingredients. Other reasons may include the less rigorous manure management in dry cow pens compared to close-up pens, such as, higher frequency of raking pens and fence line manure cleaning in freestall pens. Dry pens also tend to be further away from the parlor which may be closer to the lagoon or surrounding crop land if applicable.

Future studies are needed to investigate the causal association between bunching and stable flies and the impact of management on the stable fly to minimize the economic losses due to bunching in dairy cows. Despite the large number of dairies in our study, external generalizability of our findings to dairies that may differ in their management practices and exposures elsewhere may be limited. For example, facility designs and animal management practices may vary among states or by geographic regions. The resting and attacking behavior of the stable fly (attack times, resting times and proximity of resting locations to cows) and its association with bunching should be studied in more detail. In addition, our study did not address the prevention and control measures as well as the economic impact of bunching on cow productivity and welfare. It also should be noted that our study does not reflect necessarily bunching in beef cattle given differences in management and housing of beef cattle.

## Conclusions

Bunching of dairy cows in the study dairies differed according to the stable fly count, facility design, dairy surroundings and managemental factors including feeding and manure management. To reduce cow bunching it is recommended to apply appropriate measures to control stable flies, clean the fence line manure, reduce the crops around the cow pens and reduce the use of wet distiller grains and molasses in the ration of dairy cows during May and June.

## Supporting information

S1 File Survey for the dairy management practices between April and July, 2017. (DOCX)

S2 File Raw data. (XLSX)

S1 Video (MP4)
