# Correction: Risk factors affecting dairy cattle protective grouping behavior, commonly known as bunching, against *Stomoxys calcitrans (L*.*)* on California dairies

**DOI:** 10.1371/journal.pone.0235775

**Published:** 2020-07-01

**Authors:** Wagdy R. El Ashmawy, Deniece R. Williams, Alec C. Gerry, John D. Champagne, Terry W. Lehenbauer, Sharif S. Aly

The images for Figs 2, 3, 4, 5, and 6 are incorrectly switched. The image that appears as Fig 2 should be Fig 3, the image that appears as Fig 3 should be Fig 4, the image that appears as Fig 4 should be Fig 5, the image that appears as Fig 5 should be Fig 6, and the image that appears as Fig 6 should be Fig 2. The figure captions appear in the correct order. The authors have provided corrected versions here.

**Fig 2:**
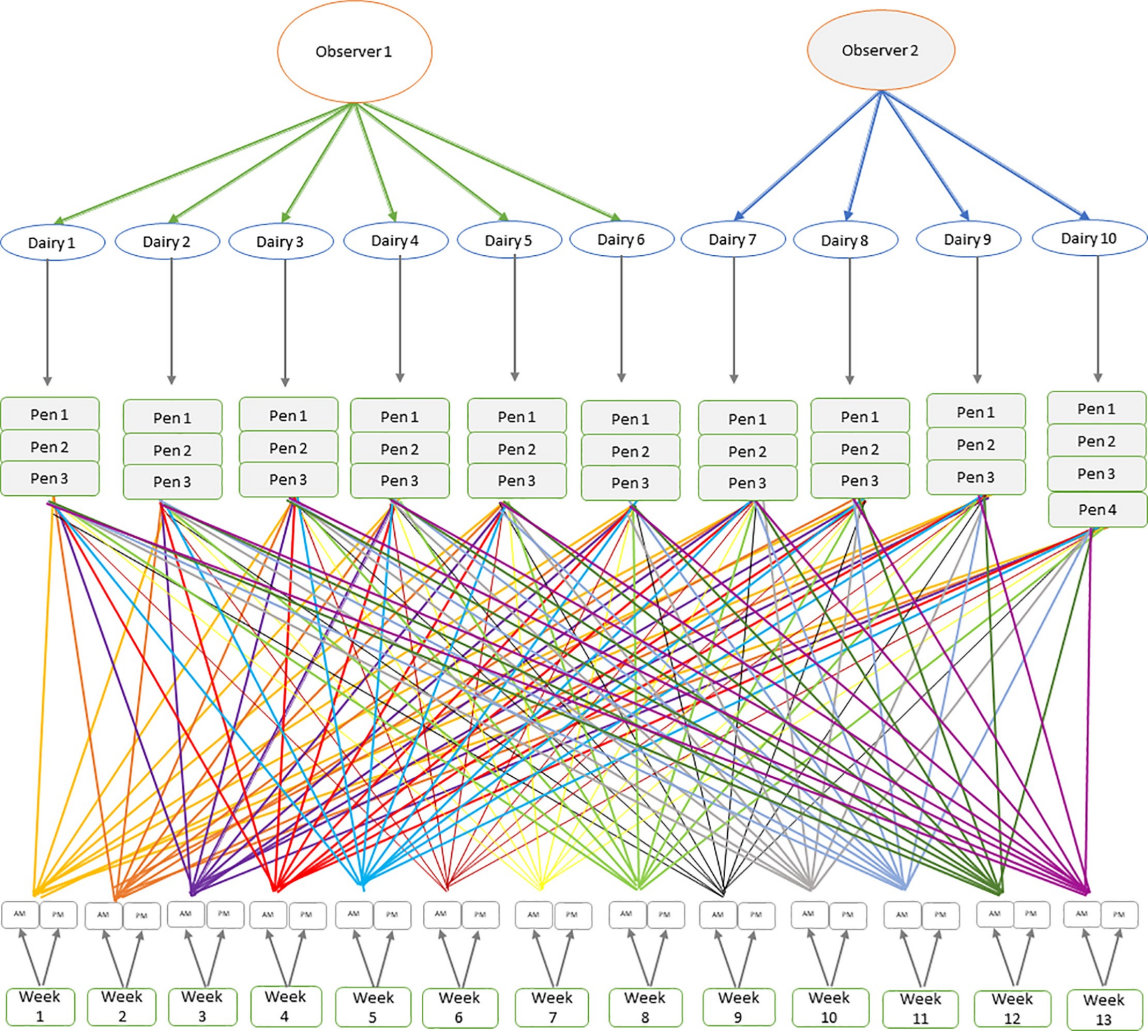
Schematic presentation of a longitudinal study of cow bunching using cow leg fly count at the pen level. Pens were nested within dairies, which were nested within observers. For example, nesting implies that pen 1 in dairy 1 differs than pen 1 in other dairies. Pens were also crossed by time of day which were nested within week. For example, crossing implies that the AM bunching observations completed at the same morning on all the pens in all the dairies.

**Fig 3:**
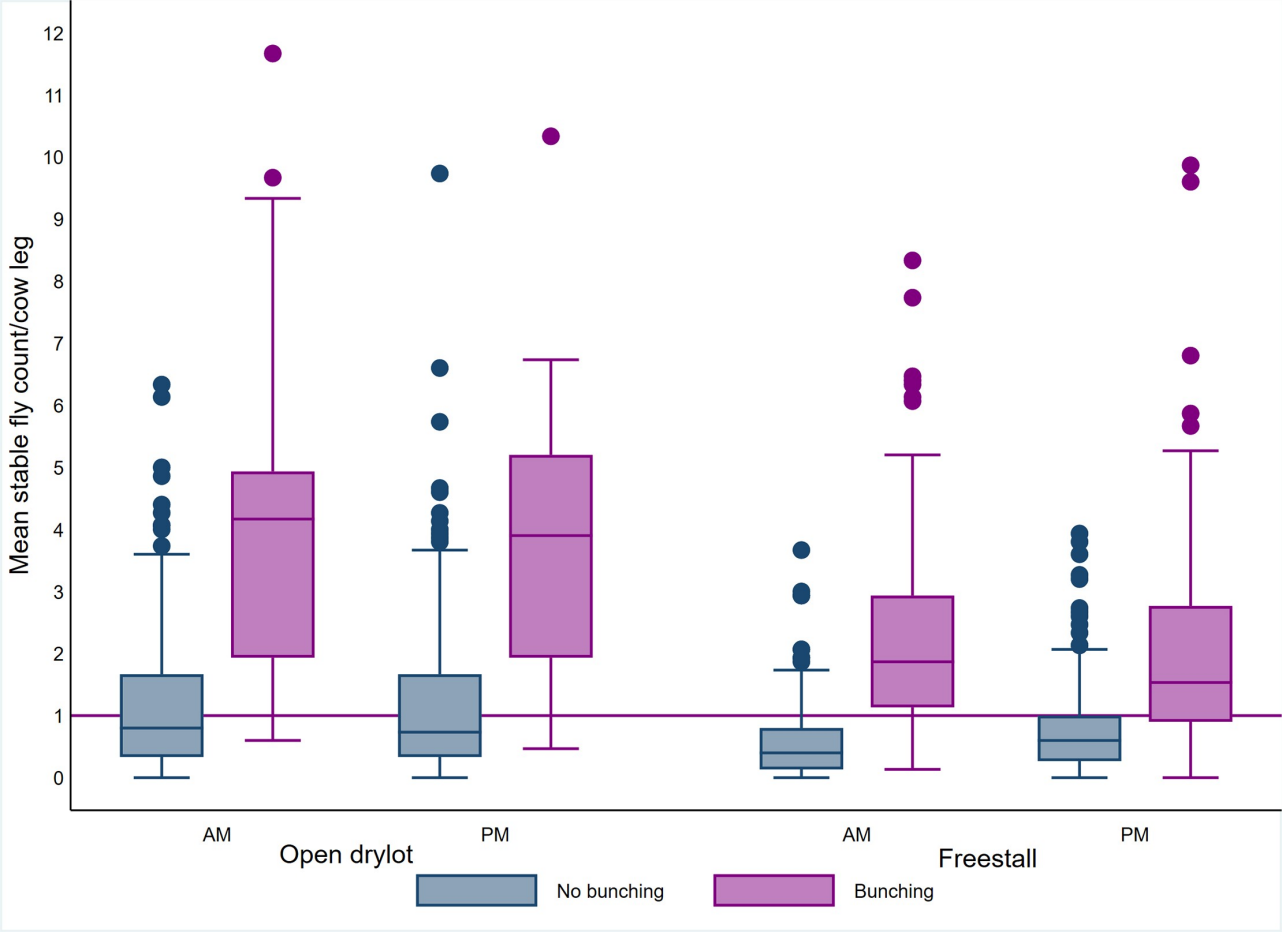
Boxplot of average stable fly count on cow leg by bunching, time of day and pen design. The upper and lower horizontal lines of the box represent the 25^th^ and 75^th^ percentiles respectively, the horizontal midline represents the median, the upper and lower horizontal whiskers lines are the upper and lower limits and the dots are the outliers.

**Fig 4:**
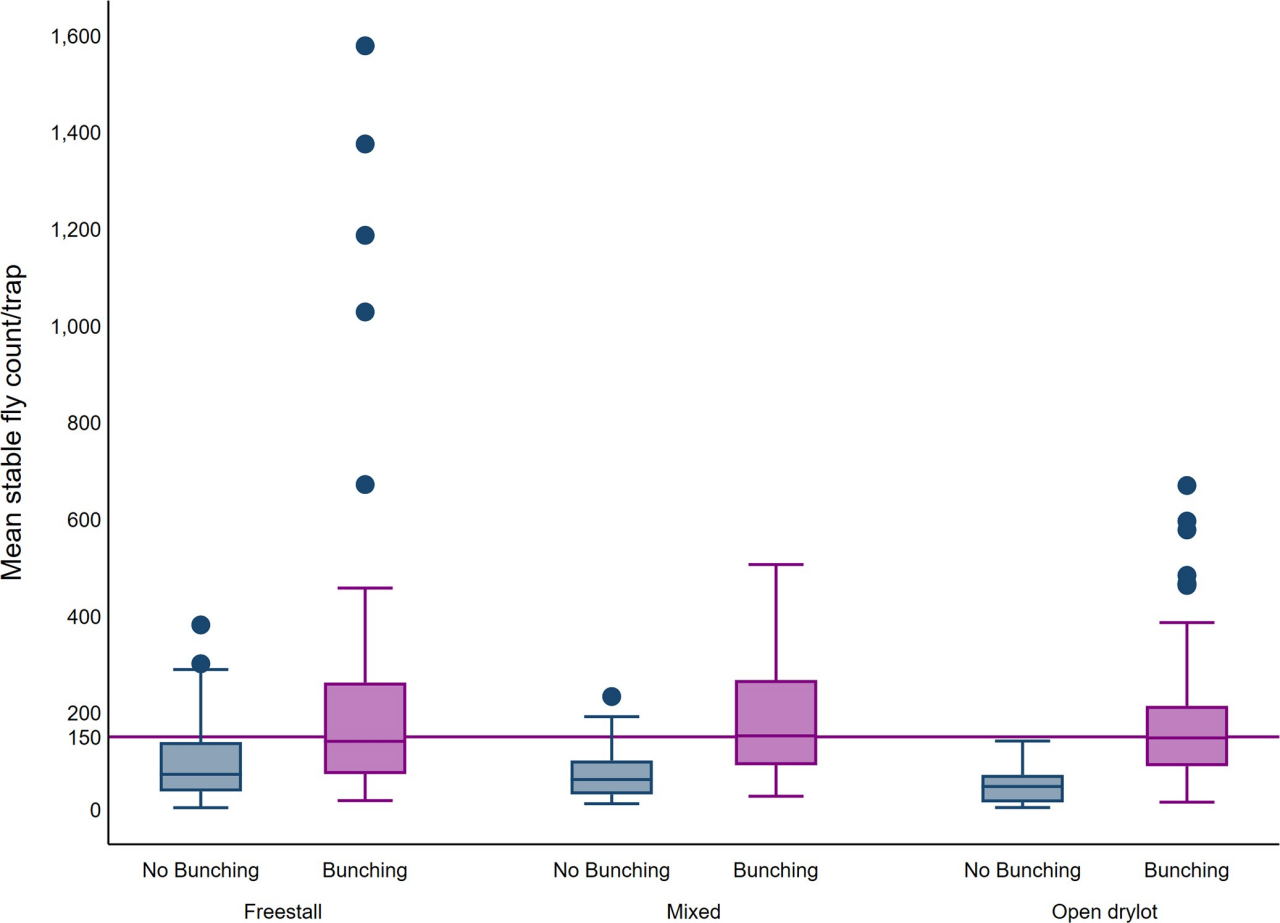
Boxplot of average trap count, bunching and farm design. The upper and lower horizontal lines of the box represent the 25^th^ and 75^th^ percentiles respectively, the horizontal midline represents the median, the upper and lower horizontal whiskers lines are the upper and lower limits and the dots are the outliers.

**Fig 5:**
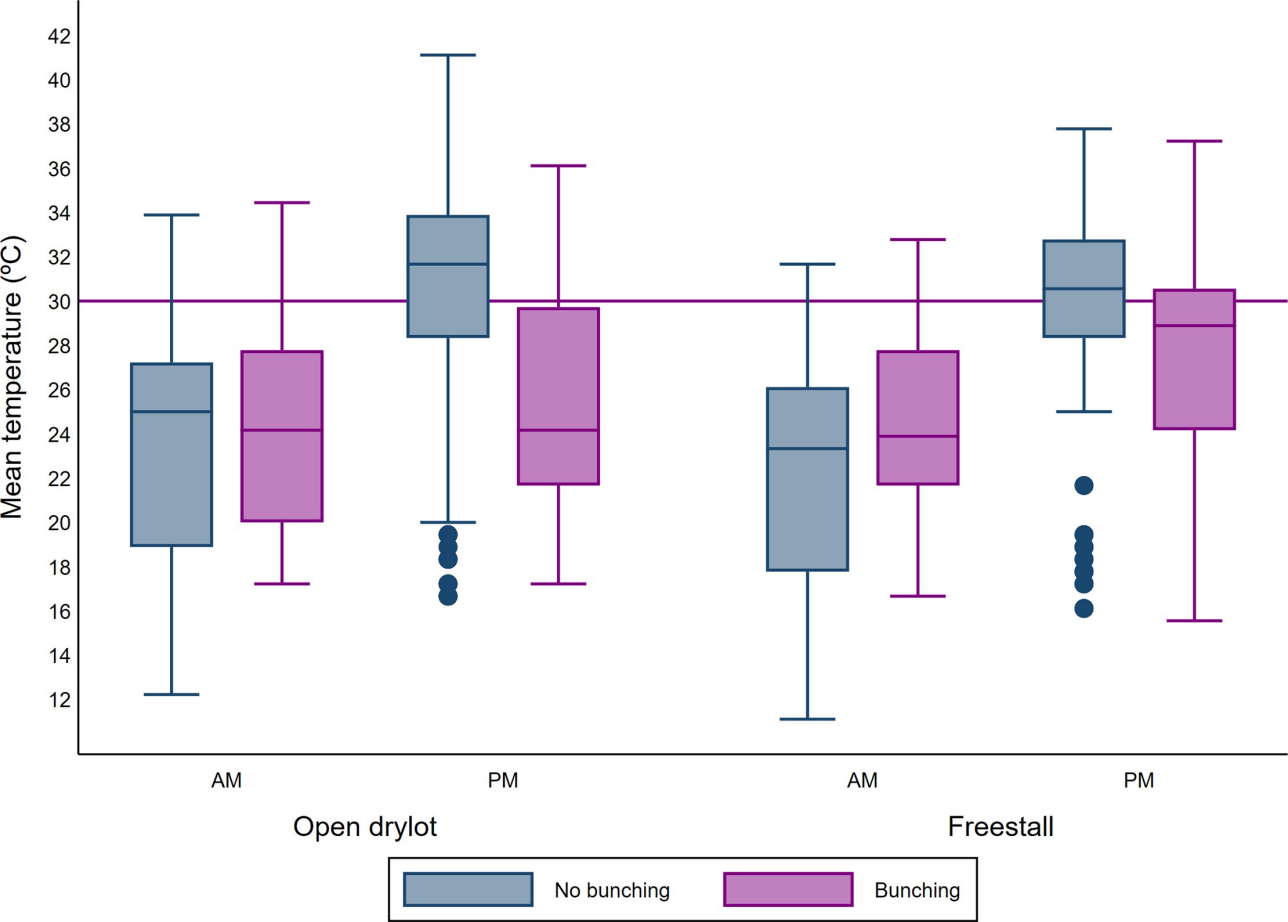
Boxplot of mean ambient temperature(°C) during AM and PM counts and bunching according to the pen design. The upper and lower horizontal lines of the box represent the 25^th^ and 75^th^ percentiles respectively, the horizontal midline represents the median, the upper and lower horizontal whiskers lines are the upper and lower limits and the dots are the outliers.

**Fig 6:**
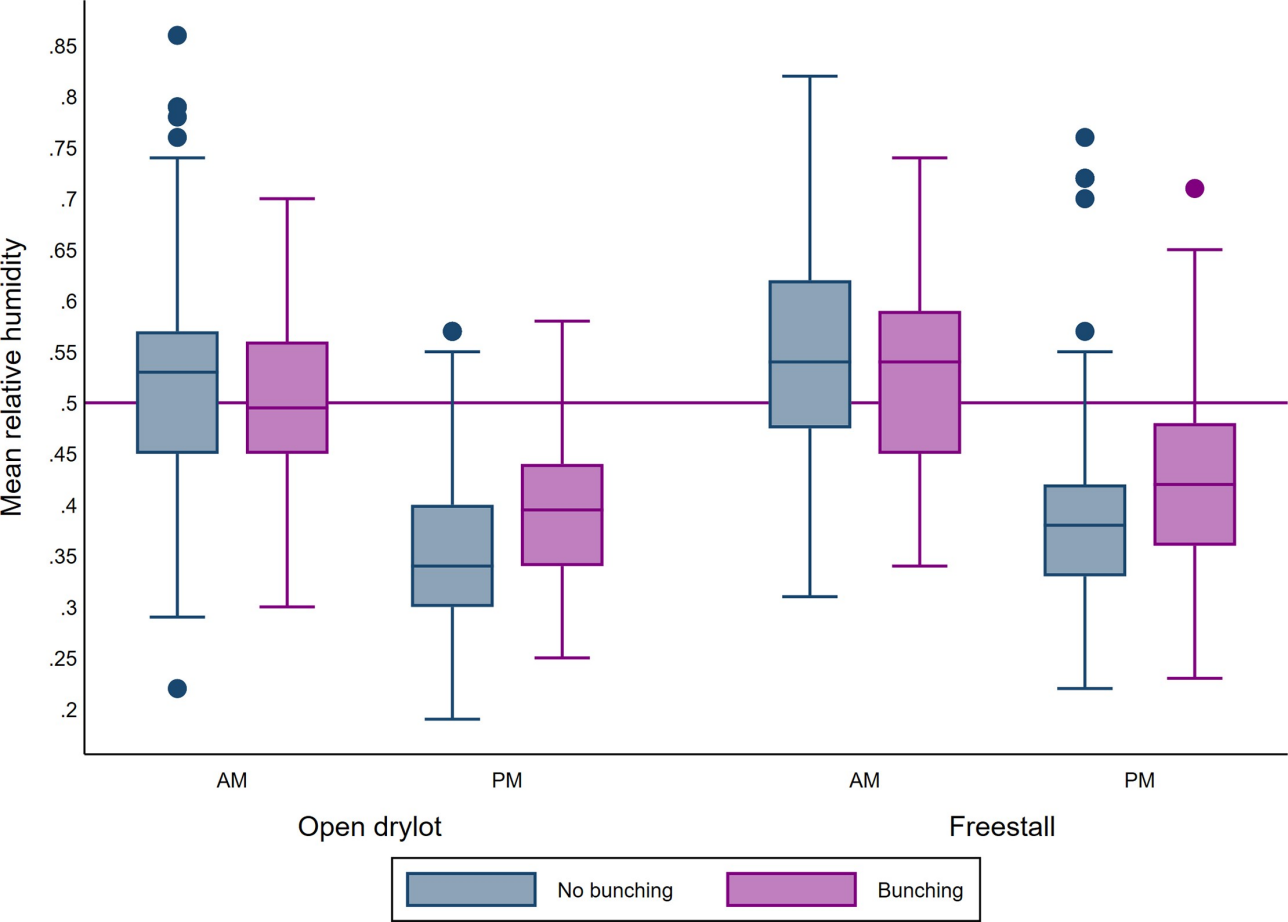
Boxplot of mean relative humidity during AM and PM counts and bunching according to the pen design. The upper and lower horizontal lines of the box represent the 25^th^ and 75^th^ percentiles respectively, the horizontal midline represents the median, the upper and lower horizontal whiskers lines are the upper and lower limits and the dots are the outliers.
