# Supplementary material for: Risk factors affecting dairy cattle protective grouping behavior, commonly known as bunching, against Stomoxys calcitrans (L.) on California dairies
Source: PLoS One. 2019 Nov 7;14(11):e0224987. doi: 10.1371/journal.pone.0224987 (PMC6837549; doi:10.1371/journal.pone.0224987)
Supplement: S1 File — (DOCX) [file pone.0224987.s001.docx]

**S1 Appendix:**
This survey refers to the current and planned management of your cattle between April and July, 2017.

**Herd Information: Date: ___________________**

| 1. Farm Name: ________________________________________________________________________________ 2. Owner’s Name: ______________________________________________________________________________ | | | | |
| --- | --- | --- | --- | --- |
| 1. Address: Street ___________________________________ City ______________ State ______ Zip ___________ | | | | |
| 1. Name and occupation of the person surveyed:   ________________________________________  ________________________________________ | | 1. Contact Information:   Phone Number: ____________________________  Email: ___________________________________  Communication Preference:    Phone call Text Email | | |
| 1. Do you give us permission to take a backup of your DHI and feed management software at the start and end of the study? Yes No | |  |  |  |
| 1. Breeds of cows in your herd:   Holstein Jersey Cross Other ______________  % ( ) ( ) ( ) ( ) | | 1. How many lactating cows do you have? | | |
|  |  | 1. What is your rolling herd average milk production? | | |
| 1. Which of these growing animals are raised on this facility? Calves Heifers Springers | | | | |
| 1. How often do you milk your cows? | 2X | | 3X | 4X |
| Pens (please list): |  | |  |  |

**Facility Design:** (list pen numbers)

| Pen Type | Lactating Cow Pens | Dry Cow Pens | Close-up Cow Pens |
| --- | --- | --- | --- |
| Freestall with exercise lot |  |  |  |
| Freestall without exercise lot |  |  |  |
| Open/Dry Lot |  |  |  |
| Bedded Pack |  |  |  |
| Other: |  |  |  |

**Management Factors:**

Bedding and Manure Management:

1. How do you maintain your facilities for the lactating cows?

| Free-stall | Dirt corrals | Feed lane |
| --- | --- | --- |
| **Cleaning**  Rake: _______ daily/weekly/monthly  Manually remove the fecal pats:    _______ daily/weekly/monthly | **Cleaning**  Rake: _______ daily/weekly/monthly  Scrape: _______ daily/weekly/monthly | **Cleaning**  Flush: _______ daily/weekly/monthly  automatic/manual  Scrape: _______ daily/weekly/monthly  Vacuum: _______ daily/weekly/monthly  Other: __________________________  _________________________________  _________________________________  _________________________________  _________________________________  _________________________________  _________________________________  _________________________________ |
| **Bedding**    Refill: _______ daily/weekly/monthly    Dried manure Sand    Other: ______________________  ______________________________  ______________________________  ___________________________  ______________________________ | **Bedding**  Bedding: _______ daily/weekly/monthly  What do you bed with?  Almond shell Straw  Dried manure Sand  Rice hulls Other:  ______________________________  ______________________________ |  |

1. Does your facility maintenance differ in the dry cow pens?  Yes  No

Cleaning: __________________________________________________________________________________

__________________________________________________________________________________________

__________________________________________________________________________________________

Bedding: ___________________________________________________________________________________

__________________________________________________________________________________________

__________________________________________________________________________________________

1. Does your facility maintenance differ in the close-up pens?  Yes  No

Cleaning: __________________________________________________________________________________

__________________________________________________________________________________________

__________________________________________________________________________________________

Bedding: ___________________________________________________________________________________

__________________________________________________________________________________

__________________________________________________________________________________

1. Does your facility maintenance differ in the winter versus summer?  Yes  No

Cleaning: __________________________________________________________________________________

__________________________________________________________________________________________

__________________________________________________________________________________________

Bedding: ___________________________________________________________________________________

__________________________________________________________________________________

__________________________________________________________________________________

1. Do you clean manure from the fencelines of your pens?  Yes, _______ daily/weekly/monthly/annually  No

If yes, how? ________________________________________________________________________________

__________________________________________________________________________________________

__________________________________________________________________________________________

1. How do you store the solid manure?  Covered manure piles  Uncovered manure piles Compost

 Other: ___________________________________________________________________________________

___________________________________________________________________________________________

1. How often do you remove solid manure from your facility? ____________________________________________
2. When was the last time that you removed solid manure from your facility? _______________________________

__________________________________________________________________________________________

1. How many lagoons do you have on your farm? ____________________________________________________
2. Is the waste water applied to surrounding crop fields?  Yes, _______ daily/weekly/monthly  No

If yes, to how many acres approximately? How is the water applied- flooding, sprinkler irrigation, other?

__________________________________________________________________________________________

__________________________________________________________________________________________

__________________________________________________________________________________________

If no, how is waste water removed from the dairy? __________________________________________________

__________________________________________________________________________________________

__________________________________________________________________________________________

__________________________________________________________________________________________

Commodity and Feed Bunk Management:

1. Do you have on-site commodity storage? Yes No
2. Do you store your silage on-site? Yes No
3. If yes, how? Drive-over piles Concrete sided bunkers Steep sided piles Silage bags
4. What by-products do you feed?

Wet distiller’s grain Fruit by-products Vegetable by-products Almond hulls Cottonseed

Other: ___________________________________________________________________________________

__________________________________________________________________________________________

1. What liquids do you add to your rations?
   1. Lactating Cows

Water Molasses Whey None Other _______________________________________

- 1. Dry Cows

Water Molasses Whey None Other _______________________________________

- 1. Close-up cows

Water Molasses Whey None Other _______________________________________

1. How many times per day do you feed your cows? (List pen numbers)

| Times/Day | Lactating Cow Pens | Dry Cow Pens | Close-up Cow Pens |
| --- | --- | --- | --- |
| 1 |  |  |  |
| 2 |  |  |  |
| 3 |  |  |  |
| 4 |  |  |  |
| 5 |  |  |  |
| 6 |  |  |  |
| >6 |  |  |  |

1. How many times per day do you push up feed to your cows? (List pen numbers)

| Times/Day | Lactating Cow Pens | Dry Cow Pens | Close-up Cow Pens |
| --- | --- | --- | --- |
| 1 |  |  |  |
| 2 |  |  |  |
| 3 |  |  |  |
| 4 |  |  |  |
| 5 |  |  |  |
| 6 |  |  |  |
| >6 |  |  |  |

1. Do you remove feed refusals from the bunks? Yes, ______ daily/weekly No
   1. If yes, do you feed the refusals to other cattle? Yes No
      1. If yes, to which animals/pens do you feed the refusals? _________________________________

_____________________________________________________________________________

_____________________________________________________________________________

- - 1. If no, what do you do with the feed refusals? _________________________________________

_____________________________________________________________________________

_____________________________________________________________________________

1. Do you remove the accumulated feed material from the top of the concrete feed curb?

Lactating Cows Yes, ______ daily/weekly/monthly No

Dry Cows Yes, ______ daily/weekly/monthly No

Close-up Cows Yes, ______ daily/weekly/monthly No

Cow Cooling:

1. Do you use soakers to cool your cows?

**Lactating Cows** Yes, automatic/manual No

1. If yes, at what ambient temperature do the soakers turn on? _______________________________________
2. What is the time-on/time-off cycle of the soakers? _______________________________________________

**Dry Cows** Yes, automatic/manual No

1. If yes, at what ambient temperature do the soakers turn on? _______________________________________
2. What is the time-on/time-off cycle of the soakers? _______________________________________________

**Close-up Cows** Yes, automatic/manual No

1. If yes, at what ambient temperature do the soakers turn on? _______________________________________
2. What is the time-on/time-off cycle of the soakers? _______________________________________________
3. Do you use fans to cool your cows?

**Lactating Cows** Yes, automatic/manual, feed lane/resting area No

1. If yes, at what ambient temperature do the fans turn on? __________________________________________
2. What is the time-on/time-off cycle of the fans? __________________________________________________

**Dry Cows** Yes, automatic/manual, feed lane/resting area No

1. If yes, at what ambient temperature do the fans turn on? __________________________________________
2. What is the time-on/time-off cycle of the fans? __________________________________________________

**Close-up Cows** Yes, automatic/manual, feed lane/resting area No

1. If yes, at what ambient temperature do the fans turn on? __________________________________________
2. What is the time-on/time-off cycle of the fans? __________________________________________________

**Bunching:**

1. Did you observe bunching on your farm last year? Yes No
   1. If yes, which months? __________________________________________________________________

____________________________________________________________________________________

- 1. At what time of the day? ________________________________________________________________

____________________________________________________________________________________

- 1. In which pens? _______________________________________________________________________

____________________________________________________________________________________

1. If bunching was observed, what were its effects on your farm?
2. On milk production: _____________________________________________________________________

_____________________________________________________________________________________

_____________________________________________________________________________________

_____________________________________________________________________________________

1. On dry matter intake: ____________________________________________________________________

_____________________________________________________________________________________

_____________________________________________________________________________________

_____________________________________________________________________________________

1. Other: ________________________________________________________________________________

_____________________________________________________________________________________

_____________________________________________________________________________________

_____________________________________________________________________________________

1. If bunching was observed, list the causes of bunching on your farm: _______________________________

__________________________________________________________________________________________

__________________________________________________________________________________________

__________________________________________________________________________________________

**Calves: If you do not raise calves onsite, please skip this section**

1. How do you rear your calves? Individually In groups
2. What kind of calf hutches do you have? Wooden “California” hutches Metal hutches Plastic hutches Other: ___________________________________________________________________________________

1. Do the hutches have an exercise area? Yes No
2. Are the hutches raised? Yes No
3. If yes, do you flush under the hutches? Yes, fresh water/recycled water No
4. If yes, how many times? _______ daily/weekly/monthly
5. Do you remove calf grain refusals? Yes, _______ daily/weekly No
6. If yes, what do you do with them? ____________________________________________________________

_______________________________________________________________________________________

_______________________________________________________________________________________

_______________________________________________________________________________________

**Adult Fly Control:**

1. Do you have a fly control program on your farm?  Yes  No
2. Do you hire a company to assist with the fly control program?  Yes  No
3. If yes, what is the name of the company and may we contact them to inquire about the services they provide? __________________________________________________________________________________________

__________________________________________________________________________________________

1. What services do they provide? _________________________________________________________________

__________________________________________________________________________________________

__________________________________________________________________________________________

__________________________________________________________________________________________

1. How often do they provide these services? _______ daily/weekly/monthly/as needed
2. Including services provided by a contracted fly control company, what additional fly control methods do you use on your lactating cows?

Fly traps: _______ daily/weekly/monthly/as needed, Name of product: _________________________________

____________________________________________________________________________________

Method of use: _______________________________________________________________________

____________________________________________________________________________________

Location of use: ______________________________________________________________________

Chemical insecticides: _______ daily/weekly/monthly/as needed, Name of product: _______________________

____________________________________________________________________________________

Method of use: _______________________________________________________________________

____________________________________________________________________________________

Location of use: ______________________________________________________________________

Other: _______ daily/weekly/monthly/as needed, Name of product: ____________________________________

____________________________________________________________________________________

Method of use: _______________________________________________________________________

____________________________________________________________________________________

Location of use: ______________________________________________________________________

1. What additional fly control methods do you use on your dry cows?

Fly traps: _______ daily/weekly/monthly/as needed, Name of product: _________________________________

____________________________________________________________________________________

Method of use: _______________________________________________________________________

____________________________________________________________________________________

Location of use: ______________________________________________________________________

Chemical insecticides: _______ daily/weekly/monthly/as needed, Name of product: _______________________

____________________________________________________________________________________

Method of use: _______________________________________________________________________

____________________________________________________________________________________

Location of use: ______________________________________________________________________

Other: _______ daily/weekly/monthly/as needed, Name of product: ____________________________________

____________________________________________________________________________________

Method of use: _______________________________________________________________________

____________________________________________________________________________________

Location of use: ______________________________________________________________________

1. What additional fly control methods do you use on your close-up cows?

Fly traps: _______ daily/weekly/monthly/as needed, Name of product: _________________________________

____________________________________________________________________________________

Method of use: _______________________________________________________________________

____________________________________________________________________________________

Location of use: ______________________________________________________________________

Chemical insecticides: _______ daily/weekly/monthly/as needed, Name of product: _______________________

____________________________________________________________________________________

Method of use: _______________________________________________________________________

____________________________________________________________________________________

Location of use: ______________________________________________________________________

Other: _______ daily/weekly/monthly/as needed, Name of product: ____________________________________

____________________________________________________________________________________

Method of use: _______________________________________________________________________

____________________________________________________________________________________

Location of use: ______________________________________________________________________

1. What additional fly control methods do you use on your calves?

Fly traps: _______ daily/weekly/monthly/as needed, Name of product: _________________________________

____________________________________________________________________________________

Method of use: _______________________________________________________________________

____________________________________________________________________________________

Location of use: ______________________________________________________________________

Chemical insecticides: _______ daily/weekly/monthly/as needed, Name of product: _______________________

____________________________________________________________________________________

Method of use: _______________________________________________________________________

____________________________________________________________________________________

Location of use: ______________________________________________________________________

Fly larva inhibitor: _______ daily/weekly/monthly/as needed, Name of product: ___________________________

____________________________________________________________________________________

Method of use: _______________________________________________________________________

____________________________________________________________________________________

Location of use: ______________________________________________________________________

Other: _______ daily/weekly/monthly/as needed, Name of product: ____________________________________

____________________________________________________________________________________

Method of use: _______________________________________________________________________

____________________________________________________________________________________

Location of use: ______________________________________________________________________

1. What additional fly control methods do you use at your feed storage area?

Fly traps: _______ daily/weekly/monthly/as needed, Name of product: _________________________________

____________________________________________________________________________________

Method of use: _______________________________________________________________________

____________________________________________________________________________________

Location of use: ______________________________________________________________________

Chemical insecticides: _______ daily/weekly/monthly/as needed, Name of product: _______________________

____________________________________________________________________________________

Method of use: _______________________________________________________________________

____________________________________________________________________________________

Location of use: ______________________________________________________________________

Other: _______ daily/weekly/monthly/as needed, Name of product: ____________________________________

____________________________________________________________________________________

Method of use: _______________________________________________________________________

____________________________________________________________________________________

Location of use: ______________________________________________________________________

1. What additional fly control methods do you use at your manure handling area?

Fly traps: _______ daily/weekly/monthly/as needed, Name of product: _________________________________

____________________________________________________________________________________

Method of use: _______________________________________________________________________

____________________________________________________________________________________

Location of use: ______________________________________________________________________

Chemical insecticides: _______ daily/weekly/monthly/as needed, Name of product: _______________________

____________________________________________________________________________________

Method of use: _______________________________________________________________________

____________________________________________________________________________________

Location of use: ______________________________________________________________________

Other: _______ daily/weekly/monthly/as needed, Name of product: ____________________________________

____________________________________________________________________________________

Method of use: _______________________________________________________________________

____________________________________________________________________________________

Location of use: ______________________________________________________________________

**Dairy Map:**

1. Please identify the following pens (including pen numbers) and areas on the provided map of your dairy:
   1. Lactating, dry, close-up, maternity, heifer, and hospital pens
   2. Calf hutches
   3. Milking parlor
   4. Commodity area
   5. Lagoons, manure handling and storage areas.
   6. Surrounding crops

| Pen No. | Production Type | Pen Design | Ridge Cap | Roof (1) | | | | Roof (2) | | | |
| --- | --- | --- | --- | --- | --- | --- | --- | --- | --- | --- | --- |
|  |  |  |  | Location | Material | Height | Width | Location | Material | Height | Width |
| ____ | _________  _________ | ________  ________ | Yes  No | __________  __________ | __________  __________ | __________  __________ | __________  __________ | __________  __________ | __________  __________ | __________  __________ | __________  __________ |

The following tables were completed for each pen:

| Pen No. | Feed curbs | Feed refusals | Fence line manure | wet spots |
| --- | --- | --- | --- | --- |
| ____ | Clean Dirty____________  __________________________ | Yes No, dry/wet/manure____________________  ____________________________________________ | Yes No, ___________  ______________________ | __________________________  __________________________ |

| Pen No. | Soakers | | Fans | | | Water troughs | |
| --- | --- | --- | --- | --- | --- | --- | --- |
|  | Present | Leaking | Presence &Location | Direction | Function | Leaking | Describe |
| ___ | Yes No  ____________ | Yes No  ____________________________ | Yes No  ____________ | __________  __________ | __________________  __________________ | Yes  No | ___________________________  ___________________________ |

1. Is fluid leaking around the silage pit(s)? Yes No

If yes, describe: ­­­­­­­______________________________________________________________________________________________

1. Is rotten silage noted on/around the face of the silage pit(s)? Yes No

If yes, describe: ­­­­­­­­­­­­­­______________________________________________________________________________________________

**S2 Appendix: Video** El-Ashmawy et al._Dairy cattle bunching due to stable flies.MP4
